# Assembly of *Ruminococcus flavefaciens* cellulosome revealed by structures of two cohesin-dockerin complexes

**DOI:** 10.1038/s41598-017-00919-w

**Published:** 2017-04-07

**Authors:** Pedro Bule, Victor D. Alves, Vered Israeli-Ruimy, Ana L. Carvalho, Luís M. A. Ferreira, Steven P. Smith, Harry J. Gilbert, Shabir Najmudin, Edward A. Bayer, Carlos M. G. A. Fontes

**Affiliations:** 1 CIISA – Faculdade de Medicina Veterinária, ULisboa, Pólo Universitário do Alto da Ajuda, Avenida da Universidade Técnica, 1300-477 Lisboa, Portugal; 2 Department of Biomolecular Sciences, The Weizmann Institute of Science, Rehovot, 76100 Israel; 3 UCIBIO-REQUIMTE, Departamento de Química, Faculdade de Ciências e Tecnologia, Universidade Nova de Lisboa, 2829-516 Caparica, Portugal; 4 Department of Biomedical and Molecular Sciences, Queen’s University, Kingston, ON K7L 3N6 Canada; 5 Institute for Cell and Molecular Biosciences, Newcastle University, The Medical School, Newcastle upon Tyne, NE2 4HH United Kingdom

## Abstract

Cellulosomes are sophisticated multi-enzymatic nanomachines produced by anaerobes to effectively deconstruct plant structural carbohydrates. Cellulosome assembly involves the binding of enzyme-borne dockerins (Doc) to repeated cohesin (Coh) modules located in a non-catalytic scaffoldin. Docs appended to cellulosomal enzymes generally present two similar Coh-binding interfaces supporting a dual-binding mode, which may confer increased positional adjustment of the different complex components. *Ruminococcus flavefaciens*’ cellulosome is assembled from a repertoire of 223 Doc-containing proteins classified into 6 groups. Recent studies revealed that Docs of groups 3 and 6 are recruited to the cellulosome *via* a single-binding mode mechanism with an adaptor scaffoldin. To investigate the extent to which the single-binding mode contributes to the assembly of *R*. *flavefaciens* cellulosome, the structures of two group 1 Docs bound to Cohs of primary (ScaA) and adaptor (ScaB) scaffoldins were solved. The data revealed that group 1 Docs display a conserved mechanism of Coh recognition involving a single-binding mode. Therefore, in contrast to all cellulosomes described to date, the assembly of *R*. *flavefaciens* cellulosome involves single but not dual-binding mode Docs. Thus, this work reveals a novel mechanism of cellulosome assembly and challenges the ubiquitous implication of the dual-binding mode in the acquisition of cellulosome flexibility.

## Introduction

The cellulosome is one of the most intricate nanomachines Nature has evolved. Cellulosomes combine an extensive repertoire of enzymes, including glycoside hydrolases, pectate lyases and carbohydrate esterases, into a large multi-enzyme complex (molecular mass >3 MDa) that efficiently deconstructs especially recalcitrant plant structural carbohydrates, such as cellulose and hemicellulose. Highly ordered protein-protein interactions are critical to a large array of cellular and biological processes. Thus, cellulosome assembly results from the binding of enzyme borne dockerin modules (Docs) to cohesin modules (Cohs) located in macro-molecular scaffolds (scaffoldins). Integration of enzymes into the cellulosome is believed to enhance the synergistic interactions between enzymes with complementary activities while promoting enzyme stability^1, 2^. This process is critical to the cycling of carbon between microbes, herbivores and plants. In addition, cellulases and hemicellulases are now used in several biotechnology-based industries, such as the bio-conversion of plant biomass into renewable fuels and the development of specific molecules with biomedical applications^3–5^.

**Figure 1:**
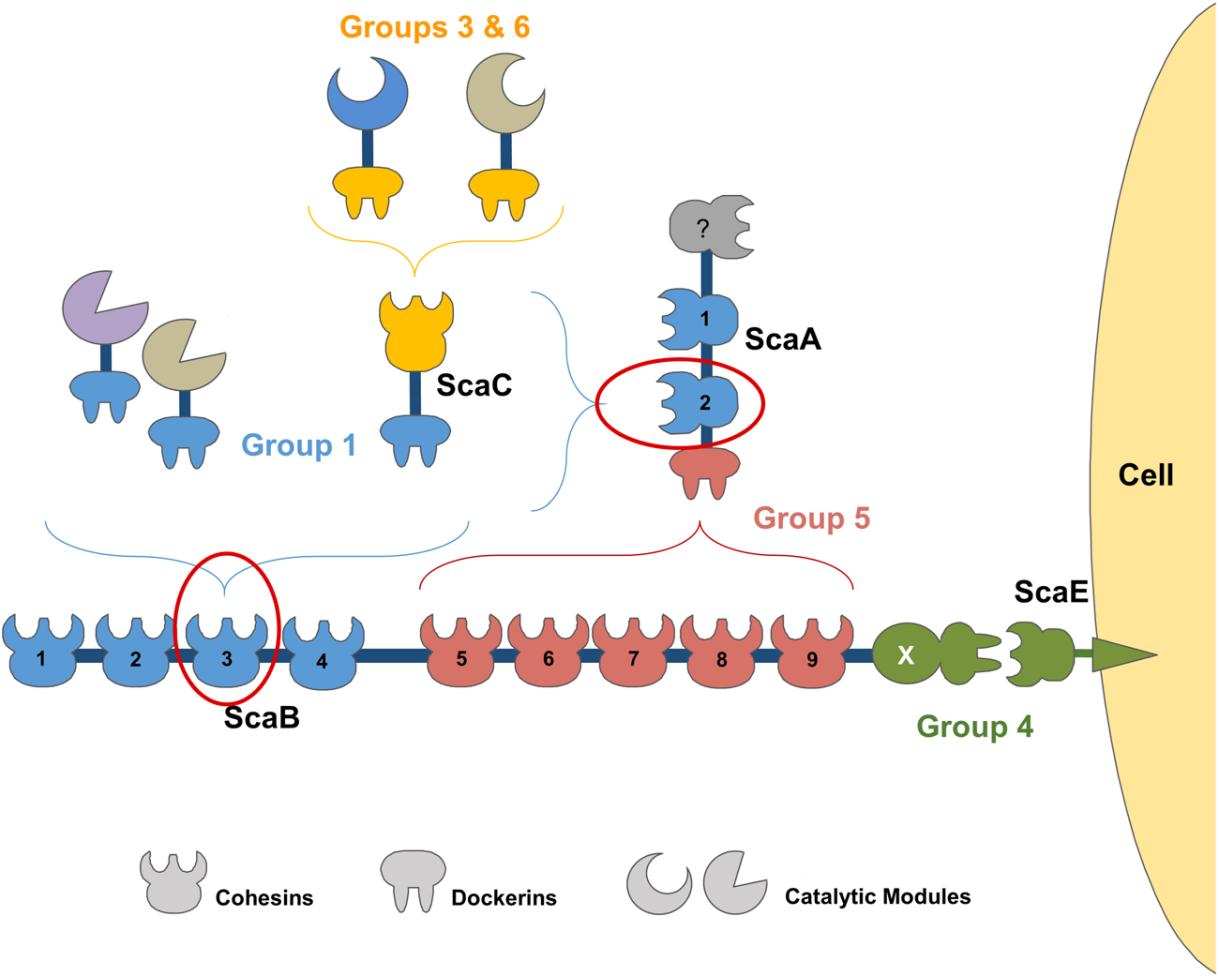
Group-specific interactions that contribute to the major cellulosome assembly in *R*. *flavefaciens* strain FD-1. The scheme is color-coded to highlight the four subgroups of cohesin-dockerin specificities: Dockerins and cognate cohesin counterparts of the different groups are marked in blue (Group 1 dockerins), yellow (Groups 3 and 6), green (Groups 2 and 4) and red (Group 5), respectively. Group 2 dockerins are truncated derivatives of group 4 and are not represented in the figure for simplification. The red ovals mark the complexes of the Group 1 interactions, whose structures are reported here.

The rumen, which essentially constitutes a large fermentation chamber in the gastrointestinal tract of ruminant mammals, is a highly competitive ecological niche colonized by symbiotic microbes that have specialized in the hydrolysis of recalcitrant carbohydrates. *Ruminococcus flavefaciens*, a Gram-positive anaerobic bacterium of the Firmicutes phylum, is one of the major cellulolytic ruminal bacteria and the only species in this microbial ecosystem that has been shown to possess a definitive cellulosome^6^. Intriguingly, the rumen houses numerous subspecies of this bacterium, each with a similar set of scaffoldins but with its own spectrum of dockerin-bearing proteins (enzymes) and cellulosome architecture^7, 8^. The genome sequence of *R*. *flavefaciens* strain FD-1 revealed the presence of 223 dockerin-containing proteins (154 of which were identified as carbohydrate-active enzymes)^8^, indicating that this bacterial nanomachine is the most complex cellulosome described to date^9^ (Fig. 1). *R*. *flavefaciens* Docs have been organized into six groups based on primary structure homology^10^. This classification was recently found to be functionally relevant^11^, with the binding of group 1 Docs to the Cohs of scaffoldins ScaA and ScaB providing the major mechanism for cellulosome assembly in *R*. *flavefaciens*. The 96 group 1 Docs have been classified in four subgroups (a to d) although the functional significance of this subdivision remains unclear. The cellulosome is tethered to the surface of *R*. *flavefaciens* through the binding of the group 4 Doc of ScaB to the Coh of the cell surface protein ScaE. A variety of other proteins were found to contain Docs that specifically interact with cell surface Cohs rather than to the cellulosomal Cohs. These Docs were classified into group 4 and group 2. Finally, hemicellulases containing group 3 or 6 Docs bind to the adaptor scaffoldin ScaC, whose group 1 Doc locks onto the Cohs in ScaA or ScaB Cohs 1–4^12, 13^. The ScaA Doc is the only member of group 5 and binds exclusively to ScaB Cohs 5–9. Figure 1 provides an overview of the organization of *R*. *flavefaciens* cellulosome.

In all clostridial cellulosomal systems described to date, such as *Clostridium thermocellum* ^14, 15^, *C*. *cellulolyticum* ^16^ and *Acetivibrio cellulolyticus* ^17^, Docs interact with their cognate Cohs through a dual-binding mode. Thus, these Docs possess the ability to bind the cognate Coh in two different orientations, by rotating ~180° with respect to its protein ligand, resulting in two different Coh-Doc conformations. The dual-binding mode results from the characteristic internal symmetry of the Doc sequence and is believed to confer additional flexibility to the macromolecular organization of cellulosomes. Recent structure/function studies, unexpectedly, showed that groups 3 and 6*R*. *flavefaciens* Docs display a single-binding mode for their target Cohs. Intriguingly, the sequence of group 1 Docs, do not seem to possess the internal symmetry required to support the dual-binding mode. This suggests that group 1 Docs may bind to their target Cohs through a single-binding mode. To test this hypothesis, we determined the X-ray crystal structure of two *R*. *flavefaciens* group 1 Docs, Doc1a and Doc1b, in complex with a ScaB (CohScaB3) and a ScaA Coh, respectively. These structures together with comprehensive biochemical analyses suggest that integration of a large repertoire of enzymes into the *R*. *flavefaciens* cellulosome operates through a single-binding mode.

## Results and Discussion

### Structure of *R. flavefaciens* ScaB cohesin 3 (RfCohScaB3)

In an initial attempt to understand the structural determinants of Coh-Doc specificity that orchestrate the correct assembly of *R*. *flavefaciens* cellulosome, the structure of the third Coh of ScaB, termed *Rf*CohScaB3, was solved by SAD phasing. Crystals belong to space group *P*4_1_2_1_2 with unit cell dimensions of *a* = *b* = 60.43 Å, *c* = 86.51 Å. Final data and structure-quality statistics are shown in Table S1. *Rf*CohScaB3 displays an elliptical structure with nine β-strands, which form two β-sheets aligned in an elongated β-barrel that displays a classical “jelly-roll fold” (Figure S1A). The two sheets comprise β-strands 9, 1, 2, 7, 4 on one face and β-strands 8, 3, 6, 5 on the other face. Strands 1 and 9 align parallel to each other, thus completing the jelly-roll, while the other β-strands are antiparallel. Structural similarity search using the PDBeFold server (http://www.ebi.ac.uk/msd-srv/ssm/) revealed that the closest, functionally relevant, structural homologs of *Rf*CohScaB3 are Cohs that bind Docs appended to enzymes, although levels of sequence similarity were relatively low. They include the Cohs from *C*. *thermocellum* ScaA (PDB code 1AOH; z score of 6.4 and root mean square deviation (rmsd) of 2.3 Å over 126 aligned residues), *Pseudobacteroides cellulosolvens* ScaB (PDB code 4UMS; z-score of 6.6 and rmsd of 1.97 Å over 120 aligned residues), *C*. *cellulolyticum* ScaA (PDB code 2VN5; z-score of 6.8 and rmsd of 2.3 Å over 124 aligned residues) and *R*. *flavefaciens* ScaC cohesin in complex with *Rf*Doc3 (PDB code 5LXV; z-score of 6.9 and rmsd of 2.1 Å over 124 aligned residues). Major differences between the Coh structures were observed at β-sheet 8-3-6-5, which constitutes the protein-interacting interface (Figure S2). In particular, the ligand binding interfaces of *Rf*CohScaB3 and *Rf*CohScaC are dramatically different explaining differences in specificity as will be described below (Figure S1B). These observations suggest that *Rf*CohScaB3 displays a unique mechanism of dockerin recognition not described in other Coh-Doc complexes.

### Structure of novel *R. flavefaciens* Coh-Doc complexes

**Figure 2:**
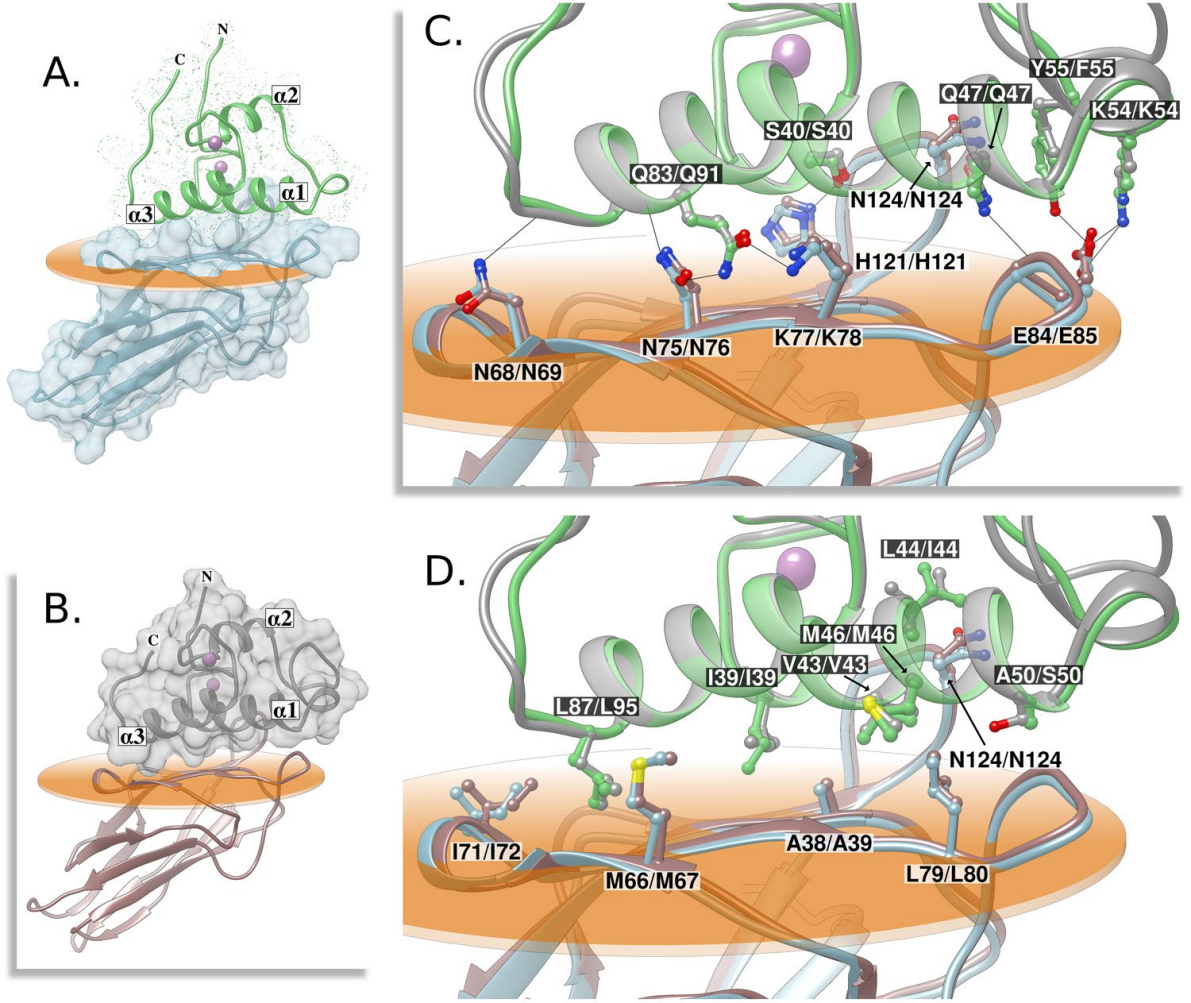
Structure and cohesin-dockerin interface of *Rf*CohScaB3-Doc1a and *Rf*CohScaA-Doc1b. (**A**) Structure of *Rf*CohScaB3-Doc1a complex with the dockerin in green and the cohesin in light blue. The dockerin N- and C- terminus and the α-helices are labeled, and a dotted molecular surface representation is shown. The cohesin blue molecular surface is represented. (**B**) Structure of *Rf*CohScaA-Doc1b complex with the dockerin in gray and the cohesin in brown, using a similar layout as in panel A. but showing instead the transparent gray molecular surface of the dockerin. (**C**) Overlay of both complexes showing the main polar interactions at the Coh-Doc interface. (**D**) Overlay of both complexes showing the main hydrophobic interactions at the Coh-Doc interface. In panels C and D. the most important residues involved in Coh-Doc recognition are depicted as ball&stick configuration, with a dark background label for the Doc residues and a light background label for the Coh residues, using the Doc1a/Doc1b and CohScaB3/CohScaA numbering. Solid black lines mark hydrogen-bonds interactions. Ca^2+^ ions are depicted as purple spheres. In all panels, the transparent orange disk marks the plane defined by the 8-3-6-5 β-sheet, where the β-strands form a distinctive dockerin-interacting plateau.

In a previous study^11^, ScaB Cohs 1 to 4 and ScaA Cohs were shown to bind specifically to group 1 Docs. In those studies, highly stable complexes were formed between *Rf*CohScaB3 and a group 1a Doc, *Rf*Doc1a, and between *Rf*CohScaA and a group 1b Doc, *Rf*Doc1b. *Rf*Doc1a is a component of a family 12 carbohydrate esterase, and *Rf*Doc1b is the C-terminal component of a family 9 glycoside hydrolase. To gain insight into the molecular mechanisms of cellulosome assembly the X-ray crystal structures of *R*. *flavefaciens* ScaA and ScaB Cohs in complex with group 1b and 1a Docs, defined as *Rf*CohScaA-Doc1b and *Rf*CohScaB3-Doc1a, respectively, were determined. The structure of *Rf*CohScaB3-Doc1a was solved by molecular replacement using the *Rf*CohScaB3 structure, described above, as the search model. The *Rf*CohScaB3-Doc1a structure includes a single copy of the heterodimer in the asymmetric unit, as well as 323 water molecules, with *Rf*Doc1a coordinating two calcium ions. The complex displays an elongated shape with overall dimensions of 40 × 35 × 66 Å and includes residues 5–141 of *Rf*CohScaB3 and residues 23–96 of *Rf*Doc1a from *R*. *flavefaciens* FD-1 (Fig. 2A). The structure of *Rf*CohScaA-Doc1b was also solved by molecular replacement using *Rf*CohScaB3-Doc1a as the search model. Like *Rf*CohScaB3-Doc1a it includes a single copy of the heterodimer in the asymmetric unit, 325 water molecules and 2 calcium ions coordinated by the Doc. *Rf*CohScaA-Doc1b is virtually identical to *Rf*CohScaB3-Doc1a and includes residues 3–143 from *Rf*CohScaA and residues 24–102 from *Rf*Doc1b (Fig. 2B). Crystal parameters for the structure of the two protein complexes and data collection statistics are summarized in Table S1. In both Coh-Doc complexes the group 1 Docs bind the 8-3-5-6 sheet of the *Rf*CohScaB3 and *Rf*CohScaA β-sandwiches, which present a predominantly flat surface. Significantly, the structures of the *Rf*CohScaB3-Doc1a and *Rf*CohScaA-Doc1b complexes were found to be very similar to each other, with an average rmsd of 0.6 Å for the two chains (Fig. 2C,D). This reflects the high degree of primary structure identity (72.7% for the Cohs and 42.2% for the Docs) shown by the two complementary protein modules.

### Structures of RfCohScaB3 and RfCohScaA in complex with their cognate Docs

The structures of *R*. *flavefaciens Rf*CohScaB3 and *Rf*CohScaA Cohs in complex with *Rf*Doc1a and *Rf*Doc1b, respectively display striking structural similarities presenting a rmsd of 0.45 Å over 136 main chain carbon atoms. As proposed above, the Doc-interacting β-sandwich face comprised β-strands 8, 3, 6 and 5 (Figure S2). No α-helices were identified in *Rf*CohScaB3 and *Rf*CohScaA Cohs (Fig. 2A,B; Figure S2), and they thus lack the distinctive α-helix connecting β-strands 4 and 5 in other bacterial Cohs as well as the large β*-*flap disrupting β-strand 8, previously observed in the *R*. *flavefaciens* ScaC group 3 Coh (ref. 12; Figure S2). The structure of *Rf*CohScaB3, whether unbound or in complex with *Rf*Doc1a, was essentially identical (rmsd ~0.37 Å). Thus, similar to previous descriptions^15, 16^, Cohs appear to be highly stable modules that do not undergo significant conformational changes upon binding to their Doc ligands.

### Structures of RfDoc1a and RfDoc1b in complex with their cognate Cohs

The structures of *Rf*Doc1a and *Rf*Doc1b in complex with *Rf*CohScaB3 and *Rf*CohScaA Cohs, respectively, comprise two α-helices arranged in antiparallel orientation extending from residues (using *Rf*Doc1a/*Rf*Doc1b numbering) Ile-39/Ile-39 to Tyr-55/Phe-55 (helix-1) and Val-76/Asn-84 to Leu-89/Leu-97 (helix-3). The two loops connecting these structural elements, in *Rf*Doc1a and *Rf*Doc1b, contain a seven-residue α-helix (helix-2) extending from Asp-59/Ala-67 to Ala-65/Gly-73, respectively (Fig. 2A,B). The tertiary structures of *Rf*Doc1a and *Rf*Doc1b adopt a similar fold with an rmsd of 0.9 Å over 68 main chain carbon atoms. Major structural differences between *Rf*Doc1a and *Rf*Doc1b Docs involve the loop extending from helix-1 and helix-2, which is longer in *Rf*Doc1b reflecting the previously identified longer linker region connecting the two duplicated repeats of group 1b Docs^10^. The overall tertiary structure of *Rf*Doc1a and *Rf*Doc1b is very similar to the enzyme-borne Docs from *C*. *thermocellum* (rmsd of ~1.4 Å, over 64 residues), *A*. *cellulolyticus* (rmsd of ~1.8 Å, over 67 residues), and *R*. *flavefaciens* group 3 Doc (Doc3) that binds the ScaC Coh (rmsd of 1.82 Å, over 59 residues). Both *Rf*Doc1a and *Rf*Doc1b contain two Ca^2+^ ions coordinated by several amino-acid residues, similar to the canonical EF-hand loop motif described in all other Docs^18^. The Ca^2+^ bound to the N-terminal repeat has a typical *n*, *n* + *2*, *n* + *4*, *n* + *11*, plus a water molecule, pattern of coordination (Figure S3). In contrast, the second Ca^2+^-binding region has an atypical coordination arrangement of *n*, *n* + *6*, *n* + *12* plus a water molecule (Figure S3).

Rf*CohScaB3-Doc1a and* Rf*CohScaA-Doc1b complex interfaces* - *Rf*Doc1a and *Rf*Doc1b helices 1 and 3 make various contacts with the surface of 8-3-6-5 β-sheets of *Rf*CohScaB3 and *Rf*CohScaA, respectively (Fig. 2C,D). Although the Coh-interacting platform is predominantly flat, the loop connecting β-strands 8 and 9 is elevated in relation to the 8-3-6-5 plane, thus remaining in close proximity to the N-terminus of helix-1 in the Doc structure. A slight elevation is also observed in the loop connecting β-strands 6 and 7, leading to a closer interaction with the C-terminus of helix-1. This means that the entire length of helix-1 of *Rf*Doc1a and *Rf*Doc1b interacts with the Coh surface, while helix-3 binds the Coh platform predominantly by the C-terminus. This contrasts with the interface of the recently described *R*. *flavefaciens Rf*CohScaC-Doc3 complex where the two Doc3 helices (helix 1 and helix 3) make similar contributions to CohScaC recognition^12^. In *Rf*CohScaC-Doc3, CohScaC’s α-helix located between β-strands 4 and 5, which is absent in Rf*CohScaB3 and* Rf*CohScaA*, is elevated in relation to the 8-3-6-5 plane allowing the entire Coh surface to be in closer proximity to both Doc α-helices. The surface electrostatic potential calculated for *Rf*CohScaB3-Doc1a and *Rf*CohScaA-Doc1b complexes reveal that the Coh- and Doc-interacting faces are predominantly uncharged (Figure S4). This is in contrast with *C*. *thermocellum* Coh-Doc complexes where a predominantly positive-charged Doc binds a negatively charged Coh, while the *Rf*CohScaC-Doc3 complex interface has an intermediate charge (Figure S4).

A large network of polar (Table 1) and hydrophobic interactions (Table S2) were identified at the *Rf*CohScaB3-Doc1a and *Rf*CohScaA-Doc1b complex interfaces (Fig. 2C,D). Although a few differences were observed, the contacts are highly conserved between the two complexes (Fig. 2C,D). The interactions between α-helix-1 of the Docs and the *R*. *flavefaciens* Cohs are dominated by Ile-39, Ser-40, Val-43, Met-46, Gln-47 and Lys-54 of *Rf*Doc1a and *Rf*Doc1b and His-121/His-121, Ala-38/Ala-39, Leu-79/Leu-80 and Glu-84/Glu-85 of *Rf*CohScaB3/*Rf*CohScaA Cohs, respectively (Fig. 2C,D). The side chains of the Ile-39/Val-43, at positions 11 and 15 of *Rf*Doc1a and *Rf*Doc1b, dominate the hydrophobic recognition of the Coh by contacting with the hydrophobic platform of the Coh created by Ala-38/Ala-39 and Leu-79/Leu-80 in *Rf*CohScaB3/*Rf*CohScaA, respectively. The highly hydrophobic character of α-helix-1 interaction is reinforced by the contacts established by Leu-44/Ile-44, Met-46/Met-46 and Ala-50/Ser-50 of *Rf*Doc1a/*Rf*Doc1b with *Rf*CohScaB3/*Rf*CohScaA Leu-79/Leu-80 and the aliphatic region of Asn-124 side-chain. The hydrogen bond network established by α-helix 1 is dominated by the interaction of Ser-40, Gln-47 and Lys-54 with His-121/His-121, Asn-124/Asn-124 and Glu-84/Glu-85 of *Rf*CohScaB3/*Rf*CohScaA, respectively. The two Docs are less conserved at the C-terminus of helix-1 and this generates differences in the interaction with the Coh. Thus, *Rf*Doc1a establishes an extra hydrogen bond between Tyr-55 Oη (Phe-55 in *Rf*Doc1b) and Oδ2 of *Rf*CohScaB3 Glu-84. In addition, the longer loop connecting helices 1 and 2 in *Rf*Doc1b allows the carbonyl of His-63 to form a hydrogen bond with Asn-124 Nδ2 of *Rf*CohScaA. In α-helix-3 the contacts are dominated by the important salt bridges established between Nε2 and Oε1 of Gln-83/Gln-91 of *Rf*Doc1a/*Rf*Doc1b with Oδ1 of Asn-75/Asn-76 and Nζ of Lys-77/Lys-78 of *Rf*CohScaB3/*Rf*CohScaA Cohs. In addition, the side chains of Leu-87/Leu-95 of *Rf*Doc1a/*Rf*Doc1b occupy the hydrophobic pocket created by Gly-73/Gly-74, Ile-71/Ile-72 and the aliphatic portion of Met-66/Met-67 of *Rf*CohScaB3/*Rf*CohScaA Cohs. The closer proximity of the two protein partners at the C-terminus of helix-3 in *Rf*CohScaB3-Doc1a protein complex allows the formation of two extra hydrogen bonds between *Rf*Doc1a and *Rf*CohScaB3 that are absent in *Rf*CohScaA-Doc1b.

**Table 1:** Main polar contacts between *Rf*CohScaB3 and *Rf*Doc1a and *Rf*ScaACoh and *Rf*Doc1b.

Hydrogen Bonds							
	*Rf*Doc1a				*Rf*CohScaB3		
	Atom	Residue	Residue #		Atom	Residue	Residue #
	ND2	ASN	32	<>	O	ASN	124
H1	OG	SER	40	<>	ND1	HIS	121
H1	OG	SER	40	<>	ND1	HIS	121
H1	NE2	GLN	47	<>	O	GLY	83
H1	OE1	GLN	47	<>	ND2	ASN	124
	NZ	LYS	54	<>	OE2	GLU	84
	OH	TYR	55	<>	OE2	GLU	84
H3	NE2	GLN	83	<>	OD1	ASN	75
H3	O	GLN	83	<>	ND2	ASN	75
H3	OE1	GLN	83	<>	NZ	LYS	77
H3	O	CYS	86	<>	NZ	LYS	117
H3	O	LEU	87	<>	ND2	ASN	68
	***Rf*** **Doc1b**				***Rf*** **CohScaA**		
	ND2	ASN	32	<>	O	ASN	124
H1	OG	SER	40	<>	ND1	HIS	121
H1	OG	SER	40	<>	ND1	HIS	121
H1	OE1	GLN	47	<>	ND2	ASN	124
H1	NE2	GLN	47	<>	O	GLY	84
	NZ	LYS	54	<>	OE2	GLU	85
	O	HIS	63	<>	ND2	ASN	124
H3	OE1	GLN	91	<>	NZ	LYS	78
H3	NE2	GLN	91	<>	OD2	ASN	76
H3	O	LEU	95	<>	ND2	ASN	69

Table was made using the PDBePISA server and the contacts were further verified manually with Coot. Some of the dockerin residues are marked as belonging either to helix 1 (H1) or to helix 3 (H3) interfaces.

### RfDoc1a and RfDoc1b present a single Coh-binding interface

**Figure 3:**
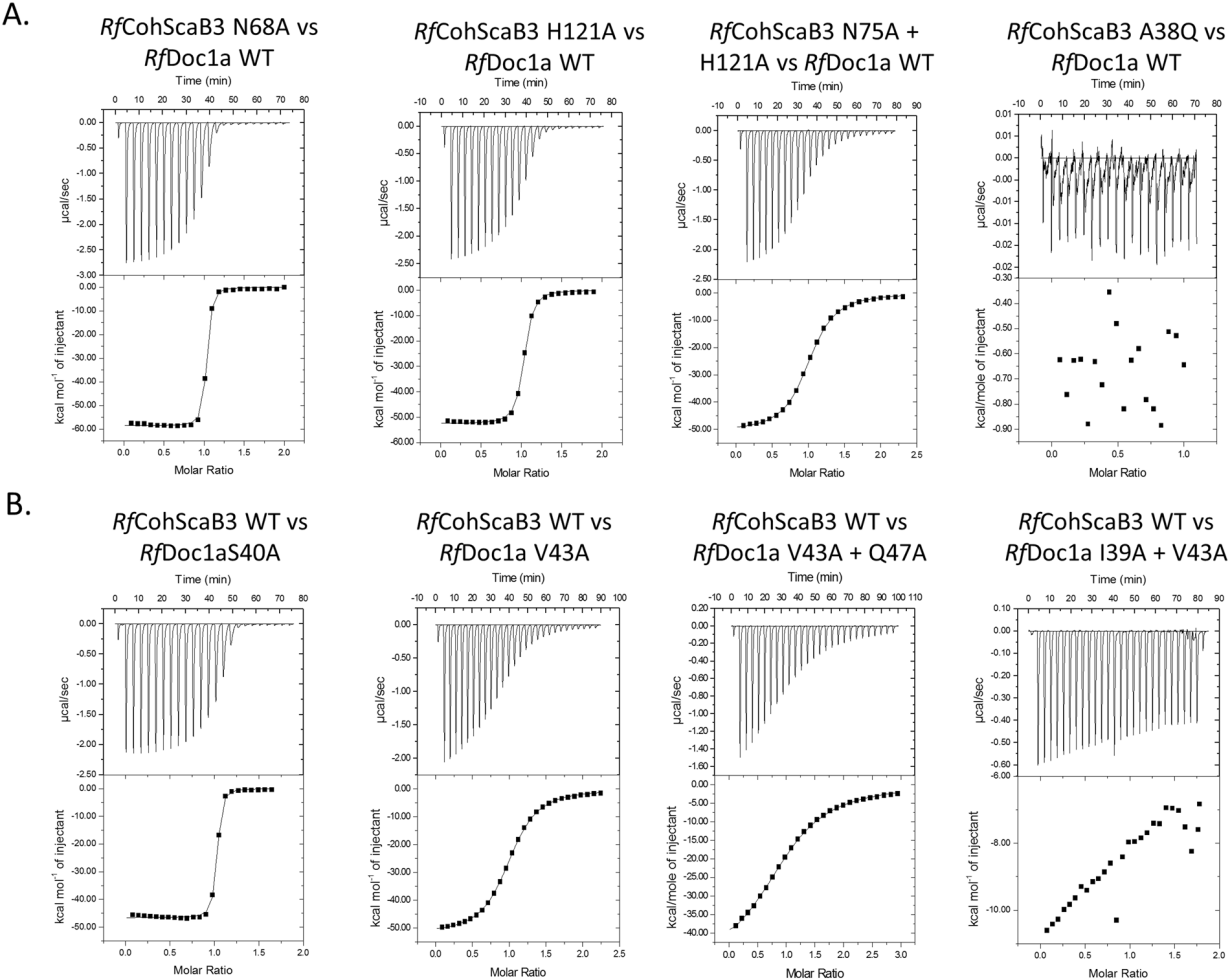
Determination of the contribution of key residues of *Rf*Doc1a and *Rf*CohScaB3 for the Coh-Doc interaction. (**A**) Representative binding isotherms of the interactions between the wild-type *Rf*Doc1a and several cohesin mutants. (**B**) Representative binding isotherms of the interactions between the wild-type *Rf*CohScaB3 and several dockerin mutants. The isotherms are arranged according to loss of function, from no loss to complete loss. The upper part of each panel shows the raw heats of binding, whereas the lower parts comprise the integrated heats after correction for heat of dilution. The curve represents the best fit to a single-site binding model. The corresponding thermodynamic parameters are shown in Table 2.

The binding thermodynamics of *Rf*Doc1a and *Rf*Doc1b to *Rf*CohScaB3 and *Rf*CohScaA were assessed by isothermal titration calorimetry (ITC) at 308 K, consistent with the approximate temperature of the rumen. The data, presented in Table 2 and exemplified in Figure S5, revealed a macromolecular association with a 1:1 stoichiometry and a *K*_a_ of ~10^7^–10^8^ M^−1^, an affinity similar to other Coh-Doc interactions. Binding was driven by changes in enthalpy with the reduction in entropy having a negative impact on affinity. The importance of *Rf*Doc1a and *Rf*CohScaB3 residues for Coh-Doc recognition was initially probed through non-denaturing gel electrophoresis (NGE) (Figure S6) and then extensively explored through ITC. The data (Table 2, Fig. 3) revealed that alanine substitutions of *Rf*Doc1a residues Ile-39 and Val-43 resulted in ~100-fold reduction in affinity of the Doc for the *Rf*CohScaB3. Complete abolition of Coh recognition resulted from the substitution of these two non-polar residues simultaneously (Table 2, Fig. 3). The alanine substitution of *Rf*Doc1a residues that participate in the hydrogen bond network with the Coh (namely Ser-40, Gln-47, Lys-54 and Gln-83) had little impact on affinity (Table 2, Fig. 3). In addition, a significant reduction in the affinity of *Rf*CohScaB3 for *Rf*Doc1a was observed following the substitution of Ala-38 with Gln and Leu-79 with Ala, the two Coh residues that create the hydrophobic environment at the *Rf*CohScaB3 platform that binds to *Rf*Doc1a. Again, the data suggest that *Rf*CohScaB3 residues that hydrogen bond with *Rf*Doc1a play a relatively small role in the binding; even when double mutants were generated the reduction in affinity was never higher than ~100 fold. Overall the data suggest that the residues that mostly influence *Rf*CohScaB3-Doc1a interaction are Ile-39 and Val-43 at helix-1 of *Rf*Doc1a and Ala-38 and Leu-79 located at the flat surface of *Rf*CohScaB3 8-3-6-5 β-sheet. Thus it seems that hydrophobic interactions play a major role in *Rf*CohScaB3-Doc1a assembly.

**Table 2:** Thermodynamics of the several interactions tested by ITC.

Cohesin	Dockerin	K_a_ M^−1^	ΔG^o^ kcal mol^−1^	ΔH kcal mol^−1^	TΔS^o^ kcal mol^−1^	N
CohScaA	Doc1bWT	2.67E7 ± 3.78E6	−10.37	−61.19 ± 0.50	−50.82	1
	Doc1aWT	5.03E8 ± 2.36E8	−12.28	−38.92 ± 0.33	−26.64	1
CohScaB3 WT	Doc1b WT	1.03E7 ± 7.63E5	−9.80	−64.94 ± 0.44	−55.13	1
	Doc1aWT	1.18E8 ± 2.00E7	−11, 23	−50.68 ± 0.29	−39.44	1
	Doc1a I39A	3.86E6 ± 7.66E4	−9.18	−57.26 ± 0.12	−48.07	1
	Doc1a S40A	1.09E8 ± 1.17E7	−11.47	−46.60 ± 0.15	−35.12	1
	Doc1a V43A	1.91E6 ± 3.02E4	−8.94	−52.08 ± 0.14	−43.14	1
	Doc1a Q47A	1.71E7 ± 6.89E5	−10.05	−48.27 ± 0.12	−38.21	1
	Doc1a K54A	1.96E7 ± 1.28E6	−10.42	−59.73 ± 0.25	−49.30	1
	Doc1a Q83A	1.54E8 ± 1.03E7	−11.71	−39.45 ± 0.83	−27.73	1
	Doc1a L87A	2.81E7 ± 1.61E6	−10.53	−59.84 ± 0.19	−49.30	1
	Doc1a I39A + V43A	Nb*	Nb*	Nb*	Nb*	Nb*
	Doc1a V43A + Q47A	4.36E5 ± 9.66E3	−7.81	−48.79 ± 0.39	−40.98	1
CohScaB3 A38Q	Doc1aWT	Nb*	Nb*	Nb*	Nb*	Nb*
CohScaB3 N68A		1.10E8 ± 1.20E7	−11.48	−58.63 ± 0.22	−47.15	1
CohScaB3 N75A		9.09E7 ± 8.96E6	−11.10	−52.70 ± 0.17	−41.60	1
CohScaB3 K77A		4.23E8 ± 7.13E7	−12.12	−57.42 ± 0.23	−45, 30	1
CohScaB3 L79A		7.59E6 ± 3.73E5	−9.71	−51.93 ± 0.20	−42.21	1
CohScaB3 E84A		3.62E7 ± 3.45E6	−10.77	−55.45 ± 0.26	−44.68	1
CohScaB3 H121A		2.66E7 ± 1.74E6	−10.41	−52.63 ± 0.18	−42.27	1
CohScaB3 N124A		5.54E7 ± 4.68E6	−10.89	−52.80 ± 0.19	−41.90	1
CohScaB3 E84A + H121A		1.65E6 ± 2.50E5	−8.62	−66.86 ± 1.49	−58.24	1
CohScaB3 N75A + H121A		2.49E6 ± 6.66E4	−8.93	−50.84 ± 0.18	−41.90	1
CohScaB3 N75A + N124A		1.86E6 ± 3.59E5	−8.85	−56.31 ± 1.56	−47.45	1
CohScaB3 N75A + E84A		2.08E7 ± 1.29E6	−10.47	−51.76 ± 0.18	−41.29	1
CohScaB3 E84A + H121A		1.65E6 ± 2.50E5	−8.62	−66.86 ± 1.49	−58.24	1
CohScaB3 E84A + N124A		1.53E7 ± 9.83E5	−10.05	−55.04 ± 0.23	−44.99	1
CohScaB3 H121A + N124A		2.28E6 ± 9.39E4	−8.84	−46.44 ± 0.24	−37.59	1

All Thermodynamic parameters were determined at 308 K.

**Figure 4:**
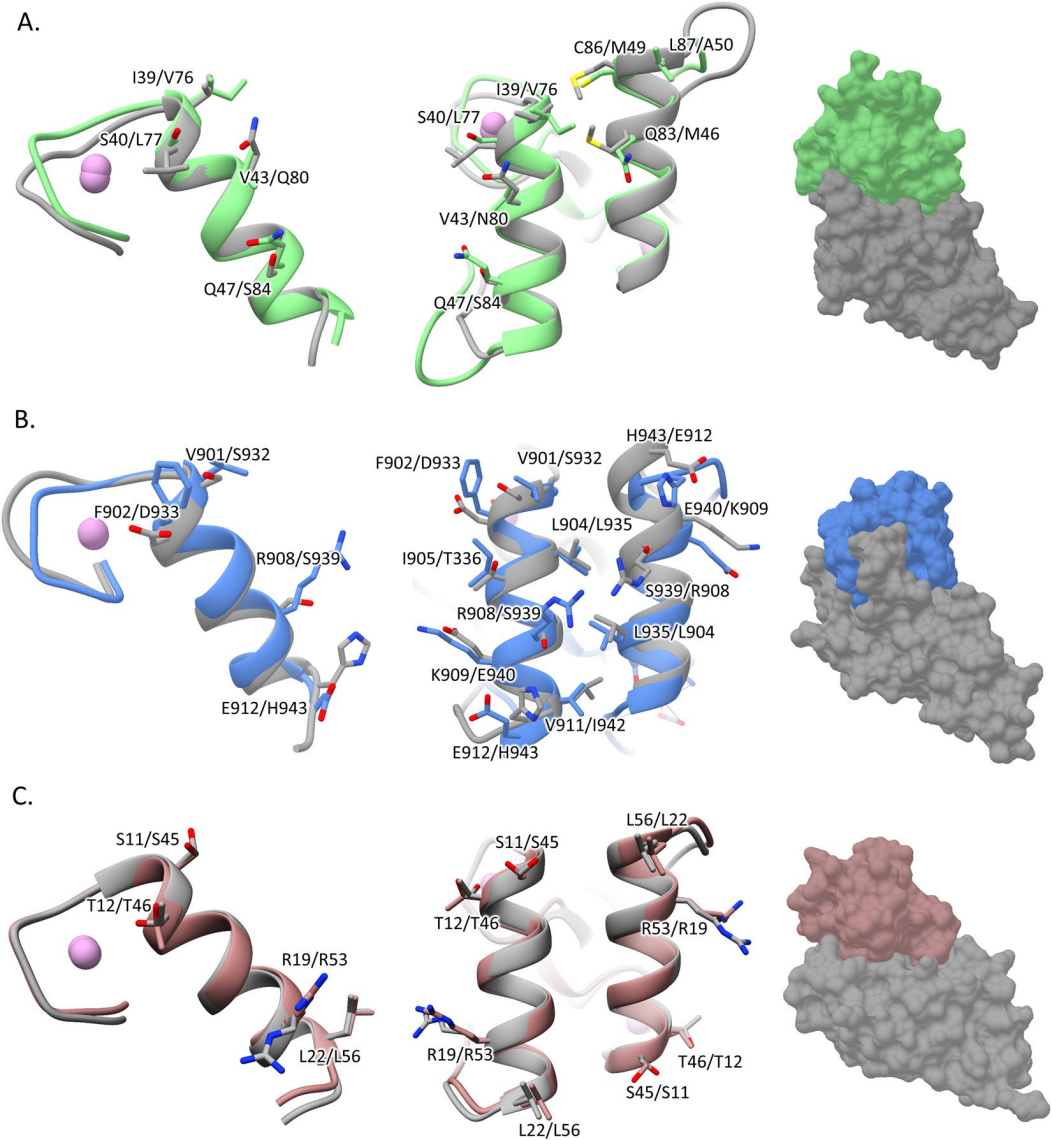
Significant differences between the two cohesin-binding interfaces do not allow the dual-binding mode of dockerins from *R*. *flavefaciens*. (**A**) *R*. *flavefaciens* Group1 Doc. (**B**) *R*. *flavefaciens* Group3 Doc. (**C**) *C*. *thermocellum* Doc. The first image of each panel shows an overlay of the N-terminal and C-terminal dockerin repeats. In all cases it is apparent that both repeats are similar at the main-chain atoms but only the *C*. *thermocellum* Doc (*C*) shows conservation in the side chains, allowing the dual-binding mode. The middle image of each panel shows a comparison of the two putative binding surfaces by overlaying the dockerins with a version of themselves rotated by 180° (in grey) and shows a lack of conservation in the key contacting residues in both *R*. *flavefaciens* dockerins (**A**,**B**). Contrary to the *C*. *thermocellum* Doc (**A**), lack of internal symmetry in Doc1a and Doc3 and the involvement of the two helices in cohesin recognition suggest that they display a single cohesin-binding platform. The final image of each panel shows the molecular surface of the several complexes, with the cohesin in grey and the dockerin in green (*Rf*Doc1a), blue (*Rf*Doc3) or pink (*C*. *thermocellum* Doc).

*Nb - No binding,

The observation that the Ile-39Ala/Val-43Ala Doc mutant did not bind to its target Coh suggests that *R*. *flavefaciens* group 1 Docs present a single-binding mode, in contrast to previous observations for the majority of Docs appended to enzymes in other organisms. When Docs present a dual-binding mode, mutation of a single or two closely positioned residues usually has no effect on affinity, as the other (duplicated) binding site is functional and can be accessed by its target Coh through a 180° rotation of the Doc. Inspection of the *Rf*CohScaB3-Doc1a structure revealed that the symmetry-related residues to Ile-39 and Val-43 (amino acids that occupy the equivalent position to Ile-39 and Val-43 when the Doc has been rotated 180°) are, respectively, Val-76 and Gln-80. While the side chain of Val-76 and Ile-39 are compatible, the bulky polar side chain of Gln-80 would be incompatible with the hydrophobic pocket in the cognate Coh that interacts with Val-43. Recent data revealed that both group 3 and group 6*R*. *flavefaciens* Docs display a single-binding mode with the ScaC Coh. The internal symmetry of *R*. *flavefaciens* group 1 and group 3 Docs when compared with the well-described dual-binding mode of enzyme Docs from *C*. *thermocellum* was therefore probed by overlaying the various structures with their 2-fold related derivatives using the Matchmaker procedure from Chimera^19^. The superposition, displayed in Fig. 4, highlights the lack of conservation in the contacting residues when the group 1 and group 3 Docs were overlayed with their 180-rotated versions. In addition to the previously mentioned changes in group 1 Docs, Ser-40 is replaced by the non-polar Leu-77 while the critical Gln-47 is replaced by Ser-84 (Fig. 4). The lack of internal symmetry is also observed in the group 3 Docs, where both α-helices 1 and 3 are involved in Coh recognition. These data, together with the extensive mutagenesis analyses presented here, suggest that group 1 Docs display a single Coh-binding platform. In contrast, the superposition of *C*. *thermocellum* enzyme Docs revealed a well-defined internal symmetry with conservation of the Coh-interacting residues when the Doc is rotated by 180°, a property that supports a dual-binding mode (Fig. 4).

### *R. flavefaciens* FD-1 Group 3 and Group 6 Docs present a non-dynamic binding mode to CohScaC

**Figure 5:**
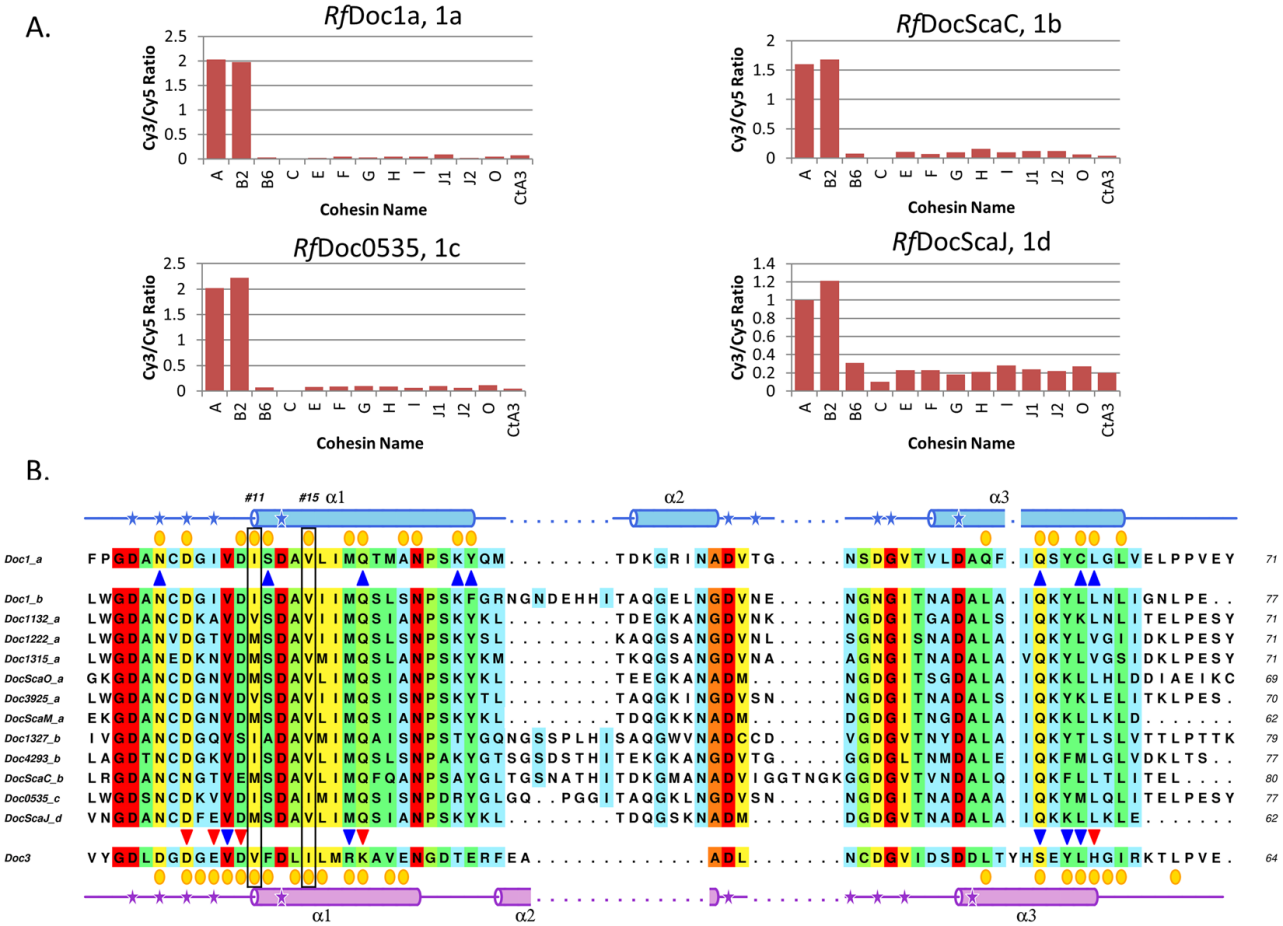
Coh-binding range and multiple sequence alignment of *R*. *flavefaciens* group 1 dockerins. (**A**) Results of Coh-Doc interactions using a cellulose microarray assay with XynDoc/CBM-Coh fusion protein pairs. Each bar graph represents the recognition profile of one dockerin from a different group 1 subgroup and 12 cohesins. The bar values correspond to the ratio between the measured Cy3 and Cy5 signals. Intensity values were calculated by Array Vision Evaluation 8.0 software and all data processing was made in Excel. (**B**) Multiple sequence alignment of *R*. *flavefaciens* group 1 Docs and group 3 Doc (Doc3). The primary sequence background is colored according to the ALSCRIPT Calcons convention, implemented in ALINE^41^: red, identical residues; orange to blue, lowering color-ramped scale of conservation. Above and below the alignment lies a cartoon representation of the secondary structure of Doc1a (blue color) and Doc3 (purple color), respectively (Coh-Doc complexes PDB codes: 5M2O and 5LXV, respectively). Also for these two Docs, the residues involved in molecular interactions with the Coh partner are represented as follows: blue triangle for hydrogen bonds, red triangle for salt bridges and yellow circles for hydrophobic contacts. Critical residues for *Rf*Doc1a/*Rf*Doc1b Coh-binding are marked with a black box, highlighting the #11 and #15 positions.

The 96 group 1 Docs identified in the proteome of *R*. *flavefaciens* FD-1 were previously organized in 4 subgroups, termed 1a to 1d^10^. *Rf*Doc1a and *Rf*Doc1b belong to group 1a (37 members) and group 1b (36 members), respectively, the most represented group 1 Docs. It was previously observed that group 1b Docs contain the longest linker region between the two Ca^2+^ repeats, although the functional significance of this remains obscure^10^. Recent data suggest that *R*. *flavefaciens* group 1 Docs display tight specificity for ScaA (Coh 1 and 2) and ScaB (Coh 1 to 4) Cohs. However, it remains unknown if the sub-classification of *R*. *flavefaciens* group 1 Docs has a functional significance. Thus, representative members of all *R*. *flavefaciens* Doc subgroups were expressed and purified. The capacity of the Docs to bind a range of representative Cohs from *R*. *flavefaciens* proteome was probed using a previously described cellulose microarray assay method^20^. The data, presented in Fig. 5 and Figure S7, revealed that all twelve Docs presented a similar binding specificity; all group 1 Docs bind tightly to CohScaA1 and CohScaB2, while not interacting with the other Cohs analyzed, including a Coh from *A*. *cellulolyticus* used as control. The primary sequences of all 13 Docs were aligned with those of group 3 Docs (Fig. 5). Initial inspection of the aligned sequences confirms, as described above, that group 1 Docs present a single-binding mode, due to a lack of internal symmetry (Figs 4 and 5). With some exceptions, strong conservation was observed in the most important residues involved in Coh recognition, namely Ile-39, Val-43, Gln-47 in helix-1 and Gln-83 and Leu-87 in helix-3 (*Rf*Doc1a residue numbering). There are, however, a few substitutions at the Ile-39 position, but these are all to non-polar residues such as Val and Met, suggesting functional conservation at this position. Taken together, the data suggest that the subgrouping of *R*. *flavefaciens* has no functional implications.

Recent studies suggest that within the *R*. *flavefaciens* proteome six Cohs, CohScaA1 and CohScaA2 and CohScaB1–4 (Fig. 1), are able to bind the 96 group 1 Docs that recruit cellulosomal enzymes to the multi-enzyme complex^11^. Residues at *Rf*CohScaB3 and *Rf*CohScaA Cohs which make direct contacts with the Doc domains, as shown in the *Rf*CohScaB3-Doc1a and *Rf*CohScaA-Doc1b structures, are mostly conserved in the four other Cohs of *R*. *flavefaciens* ScaA and ScaB scaffoldins (Figure S8). Changes that might disturb the Coh-Doc interaction are observed in CohScaB4, with the replacement of the conserved Ser-37 by a Cys (β-strand 3) and the highly conserved His-121 (β-strand 8) by a Val. The His121Val substitution would remove the hydrogen bond partner for Doc Ser-40. However, this may be compensate by the Gly126Asn change observed in the loop connecting β-strands 8 and 9 of CohScaB4, which can form the required hydrogen-bonding partner for Doc Ser-40. Thus, overall conservation in the residues involved in cellulosome assembly suggests that CohScaA1, CohScaA2 and CohScaB1-4 of *R*. *flavefaciens* will be unable to discriminate between the different group 1 Docs appended to cellulosomal enzymes. In contrast, comparison of the structure of the group 1 Coh-Doc complexes with that of the group 3 *Rf*CohScaC-Doc3 complex explains why the ScaA and ScaB Cohs cannot bind group 3 or 6 Docs, while conversely ScaC Coh is unable to recognize group 1 Docs. Other differences besides the presence of the important loop interrupting β-strand 8 in ScaC Coh, include the presence of the bulky hydrophobic side chain (usually Phe) of group 3 and 6 Docs at the critical Ser-40 position of group 1 Docs, which would make steric clashes with group 1 Cohs. Conversely, Ser-40 in group 1 Docs would not make productive interactions with the hydrophobic pocket in the ScaC Coh that is occupied by Phe side-chain in group 3 Docs.

## Conclusions

Previous structure-function studies of the cellulosomes of *C*. *thermocellum* ^14, 15^ and *C*. *cellulolyticum* ^16^ revealed that Docs used to recruit the microbial enzymes to these highly intricate multi-enzyme complexes display a dual-binding mode. In addition, recent reports revealed that the attachment of cellulosomes to the *P*. *cellulosolvens* ^21^ and *A*. *cellulolyticus* cell surface is also mediated by Docs that display a dual-binding mode^17, 22^. The structure of dual-binding mode Docs presents a 2-fold internal symmetry that allow binding to the Coh partner in two 180°-related alternate positions. The fact that Docs, in general, possess two different Coh-interacting platforms displaying identical specificities suggests that the dual-binding mode could contribute to enhance the conformational flexibility of the quaternary architecture of the highly populated multi-enzyme complex. This was supported by the observation that non-cellulosomal Docs that recruit single enzymes directly to the cell surface of *C*. *thermocellum* present a single-binding mode^23^. In addition, the Coh-Doc interaction used by *C*. *perfringens* to assemble a two-protein toxin, which is thus also not related to cellulosome assembly, was also shown to display a single-binding mode^24^. In contrast, a recent analysis of the *R*. *flavefaciens* cellulosome describes a new system in which this is not observed^12^. In this bacterium, a large repertoire of hemicellulases is appended to group 3 and 6 Docs, which specifically bind to the Coh of the adaptor scaffoldin ScaC. ScaC contains a group 1 Doc, similar to *Rf*Doc1a and *Rf*Doc1b, which interacts with ScaB and ScaA Cohs. Notably, the structure of a *R*. *flavefaciens* group 3 Doc, Doc3, in complex with CohScaC, revealed the presence of a single Coh-binding interface that involves both Doc helices^12^. Here, we extended these studies to establish if Docs displaying a single-binding mode mechanism is a generic feature of enzyme recruitment into the *R*. *flavefaciens* cellulosome. The data revealed that, similar to previously reported group 3 and 6 Docs, lack of internal symmetry in group 1 *R*. *flavefaciens* Docs generated an unconventional single protein-binding interface. This property might be widespread among all the 96 group 1 Docs, suggesting that assembly of *R*. *flavefaciens* cellulosome involves, uniquely, single-binding mode Docs. The data presented in this report questions the widely held hypothesis that the dual-binding mode mechanism provides the conformational flexibility required to degrade plant cell walls in which the topology of these composite structures varies between plants and during the degradative process. We propose that the dual-binding mode mechanism has evolved to enable rotation of the Docs in cellulosomes with a limited scaffoldin repertoire, a requirement to minimize steric clashes between the enzyme components thus increasing the number of enzyme combinations that can populate these protein complexes. The complexity of the *R*. *flavefaciens* cellulosome primary and adaptor scaffoldins reduces the steric constraints imposed by enzyme assembly obviating the need for Docs to display a dual-binding mode.

## Methods

### Gene synthesis and DNA cloning

Dockerins are inherently unstable when produced in *Escherichia coli*. To promote dockerin stability, *R*. *flavefaciens* FD-1 group 1 dockerins from protein WP_009986495 (residues 577-649) and protein WP_009982745 (residues 783-862), termed Doc1a and Doc1b, were co-expressed *in vivo* with ScaB cohesin 3 (CohScaB3) and ScaA cohesin (CohScaA), respectively. The immediate binding of the expressed dockerins to the expressed cohesins confers the necessary dockerin stabilization. The genes encoding the proteins were designed considering the optimization of codon usage to maximize expression in *E*. *coli*, synthesized *in vitro* (NZYTech Ltd, Lisbon, Portugal) and cloned into pET28a (Merk Millipore, Germany) under the control of separate T7 promoters. The dockerin-encoding genes were positioned at the 5′ end and the cohesin-encoding genes at the 3′ end of the artificial DNA. A T7 terminator sequence (to terminate transcription of the dockerin gene) and a T7 promoter sequence (to control transcription of the cohesin gene) were incorporated between the sequences of the two genes. This construct contained *Nhe*I and *Nco*I recognition sites at the 5′ end and *Xho*I and *Sal*I at the 3′ end specifically tailored to allow subcloning into pET-28a (Merk Millipore, Germany), such that the sequence encoding a six-residue His tag could be introduced either at the N-terminus of the dockerin (through digestion with *Nhe*I and *Sal*I, incorporating the additional sequence MGSSHHHHHHSSGLVPRGSHMAS at the N-terminus of the polypeptide) or at the C-terminus of the cohesin (by cutting with *Nco*I and *Xho*I, which incorporates the additional sequence LEHHHHHH at the C-terminus of the polypeptide). Thus, as a result of this strategy, two pET28a plasmid derivatives were produced for each Coh-Doc pair: one leading to the expression of dockerin with an engineered hexa-histidine tag and a second derivative where the engineered tag is attached to the cohesin. The plasmids were used to express *Rf*CohScaA-Doc1a and *Rf*CohScaB3-Doc1b protein complexes in *E*. *coli*. Recombinant Doc1a, Doc1b, CohScaA and CohScaB3 primary sequences are presented in Table S3. To produce the recombinant cohesins and dockerins individually, two distinct cloning methods were used. Digesting the previously described cohesin-tagged version of the pET28 derivatives with BglII allowed removal of the dockerin sequence. Plasmid integrity was reconstituted by re-ligating. This strategy allowed producing two novel pET28a derivatives encoding recombinant cohesins CohScaA and CohScaB3 containing C-terminal hexa-histidine tags. Dockerin-encoding genes were cloned into the pHTP2 vector (NZYtech, Lisbon, Portugal) using NZYEasy Cloning & Expression System (NZYtech, Lisbon, Portugal), following the manufacturer’s protocol. Dockerin genes were isolated by PCR using *R*. *flavefaciens* FD-1 genomic DNA as a template and the primers shown in Table S4. Recombinant dockerins encoded by the pHTP2 derivatives contained an N-terminal thioredoxin A and an internal hexa-histidine tag for increased protein stability and solubility. Sequences of all plasmids produced were confirmed by Sanger sequencing.

To identify the residues that modulate Coh-Doc specificity, several Doc1a and CohScaB3 protein derivatives were produced by site-directed mutagenesis of the pHTP2 and pET28a derivatives encoding the two genes. Site-directed mutagenesis was performed by PCR amplification using the primers presented in Table S4, which allowed the production of nine Doc1a protein derivatives (I39A, S40A, V43A, Q47A, K54A, Q83A, L87A, I39A + V43A, V43A + Q47A) and fourteen CohScaB3 protein derivatives (A38Q, N68A, N75A, K77A, L79A, E84A, H121A, N124A, E84A + H121A, N75A + H121A, N75A + N124A, N75A + E84A, E84A + N124A, H121A + N124A). Each of the newly generated gene sequences was fully sequenced to confirm that only the desired mutation accumulated in the nucleic acid.

For the cellulose microarray experiments, a system designed to fuse the Docs with a xylanase and the Cohs to a carbohydrate-binding module was selected. This allows production of highly stable and functional Cohs that can be immobilized in a cellulose-coated glass slide and Docs that can be recognized by an *α*-xylanase antibody^20^. Thus, sequences encoding the various cohesins and selected group 1 Docs were amplified from *R*. *flavefaciens* FD-1 genomic DNA by PCR, using NZYProof polymerase (NZYTech Ltd., Portugal) and the primers shown in supplemental Table S5. After gel purification, the Doc-encoding amplicons were inserted into a xylanase-Doc cassette in the pET9d plasmid after digestion with *Kpn*I and *Bam*HI and ligation with T4 ligase. The resulting expressed products consist of His-tagged Docs fused to xylanase T-6 from *Geobacillus stearothermophilus* at the N terminus of the polyhistidine tag (XynDoc). The Coh-encoding genes were cloned into a CBM-Coh cassette in pET28a after digestion with *BamH*I and *Xho*I restriction enzymes. This resulted in His-tagged Coh recombinant derivatives fused to a CBM3a from the *C*. *thermocellum* scaffoldin ScaA (CBMCoh)^25, 26^.

### Expression and Purification of Recombinant proteins

Preliminary expression screens revealed that when the hexa-histidine tag was located at the dockerin N-terminal end of both *Rf*CohScaB3-Doc1a and *Rf*CohScaA-Doc1b complexes, the expression levels of both cohesin and dockerin were higher. Tagging the cohesin resulted in the accumulation of large levels of unbound cohesin in the purification product suggesting that cohesin was expressed at higher levels than dockerins or that untagged dockerin was less stable. Thus, pET28a derivatives encoding the protein complexes formed using the tagged dockerin were subsequently used to transform *E*. *coli* BL21 (DE3) cells in order to produce *Rf*CohScaB3-Doc1a and *Rf*CohScaA-Doc1b protein complexes in large quantities. Recombinant *E*. *coli* were grown at 37 °C to an OD_600_ of 0.5. Recombinant protein expression was induced by the addition of 1 mM isopropyl *β*-D-1-thiogalactopyranoside followed by incubation at 19 °C for 16 hours. Cells were harvested by 15 min centrifugation at 5000 × *g* and resuspendend in 20 mL of immobilized-metal affinity chromatography (IMAC) binding buffer (50 mM HEPES, pH 7.5, 10 mM imidazole, 1 M NaCl, 5 mM CaCl_2_). Cells were then disrupted by sonication and the cell-free supernatant recovered by 30 min centrifugation at 15,000 × *g*. After loading the soluble fraction into a HisTrap^TM^ nickel-charged Sepharose column (GE Healthcare, UK), initial purification was carried out by IMAC in a FPLC system (GE Healthcare, UK) using conventional protocols with a 35 mM imidazole wash and a 35–300 mM imidazole elution gradient. Fractions containing the purified cohesin–dockerin complexes were buffer exchanged into 50 mM HEPES, pH 7.5, containing 200 mM NaCl, 5 mM CaCl_2_ using a PD-10 Sephadex G-25M gel-filtration column (Amersham Pharmacia Biosciences, UK). A further purification step by gel-filtration chromatography was performed by loading the Coh-Doc complexes onto a HiLoad 16/60 Superdex 75 (GE Healthcare, UK) at a flow rate of 1 ml min^−1^. Fractions containing the purified complexes were then concentrated with Amicon Ultra-15 centrifugal devices with a 10-kDa cutoff membrane (Millipore, USA) and washed three times with molecular biology grade water (Sigma) containing 0.5 mM CaCl_2_. The protein concentration was estimated in a NanoDrop 2000c spectrophotometer (Thermo Scientific, USA) using a molar extinction coefficient (ε) of 9 075 M^−1^ cm^−1^ for *Rf*CohScaB3-Doc1a and 13 075 M^−1^ cm^−1^ for *Rf*CohScaA-Doc1b. The final protein concentrations were adjusted to 40 mg.mL^−1^ for the *Rf*CohScaB3-Doc1a complex and 27 mg.mL^−1^ for *Rf*CohScaA-Doc1b, and stored in molecular biology grade water containing 0.5 mM CaCl_2_. The purity and molecular mass of the recombinant complexes were confirmed by 14% (w/v) SDS–PAGE. A similar protocol was used to produce *Rf*CohScaB3 used in the crystallization trials and its seleno-methionine derivative, except that in the latter the protein was expressed in the methionine auxotroph B834 strain of *E*. *coli*, using the growth conditions described by Ramakrishnan *et al*.^27^, and a reducing agent was added to all the buffers: 5 mM of 2-mercaptoethanol in affinity-chromatography buffers, 5 mM DTT in size-exclusion chromatography buffer and 1 mM TCEP in storage buffer. The final protein concentrations were adjusted to 47 mg.mL^−1^.

Group 1 dockerins and *R*. *flavefaciens* cohesins and their respective mutant derivatives used in native PAGE and ITC experiments were expressed as described before and purified with IMAC using nickel-charged Sepharose His GraviTrap gravity-flow columns (GE Healthcare, UK). After IMAC, the recombinant cohesin and dockerins were buffer exchanged to 50 mM HEPES pH 7.5, 0.5 mM CaCl_2_ and 0.5 mM TCEP using PD-10 Sephadex G-25M gel filtration columns (GE Healthcare, UK).

### Nondenaturing gel electrophoresis (NGE)

For the NGE experiments, each Doc variant (30 μM) was incubated in the presence and absence of 30 μM Coh for 30 min at room temperature and separated on a 10% native (lacking SDS) polyacrylamide gel. Electrophoresis was carried out at room temperature. The gels were stained with Coomassie Blue. Complex formation was detected by the presence of an additional band, usually displaying a lower electrophoretic mobility than that of the individual modules.

### Isothermal Titration Calorimetry

All ITC experiments were carried out at 308 K. The purified Doc and Coh variants were diluted to the required concentrations and filtered using a 0.45 μm syringe filter (PALL). During titrations the dockerin constructs were stirred at 307 revolutions/min in the reaction cell and titrated with 28 successive 10 μL injections of cohesin at 220 s intervals. Integrated heat effects, after correction for heats of dilution, were analyzed by nonlinear regression using a single-site model (Microcal ORIGIN version 7.0, Microcal Software, USA). The fitted data yielded the association constant (K_A_) and the enthalpy of binding (ΔH). Other thermodynamic parameters were calculated using the standard thermodynamic equation: *ΔRTlnK*_*A*_ = *ΔG* = *ΔH* − *TΔS*.

### Cellulose microarray

The cellulose microarray approach was conducted using the XynDoc/CBM-Coh fusion protein pairs, in order to evaluate cohesin-dockerin interactions by refining the method described in Barak *et al*.^20^ DNA isolation and cloning were performed as described above. The strong selective binding of the CBM to the cellulose-coated slides was used as an intrinsic purification step so that cohesins were thus applied to the glass slides as crude extracts. The dockerins were purified as described above.

Rabbit anti-XynT6 primary antibody was conjugated with fluorescent Cy3 dye and rabbit anti-CBM primary antibody with fluorescent Cy5 dye, in order to assess signal intensity and normalize with the amount of protein, respectively. Xyn-CBM fusion protein was designed, cloned and expressed in the form of crude extract, as a positive control for the Cy3- and Cy5-conjugated antibodies. For biological positive controls, pre-established interactions were included in the setup. To eliminate the possibility of any of *E*. *coli*’s background components generating a false signal, BL-21 were transformed with an empty pET28a vector, which lacks a CBM or a cohesin module. The cellulose-coated glass slides were printed with crude extracts of this negative control that were subjected to the same treatment and storage conditions.

Although protein amounts were validated on SDS-PAGE gels prior to screening, there was still printing variation resulting from the use of a hand arrayer. It was therefore necessary to estimate the ratio between the Cy3 signal intensity, which indicates the presence of XynDoc, and the Cy5 signal intensity, which stands for the amount of CBM-Coh that is present in the area of a specific spot. This was done with ‘Array Vision Evaluation 8.0’ software. Raw data were further processed in Excel to generate bar graphs.

### X-ray crystallography, Structural Determination and Refinement

Crystallization conditions were set up using the sitting-drop vapor diffusion method with a robotic nanodrop dispensing system Oryx8 (Douglas Instruments, UK). Commercial kits Crystal Screen, Crystal Screen 2, PEG Ion Screen I and II from Hampton Research (California, USA), JCSG + HT96 (Molecular Dimensions, UK) and an in-house screen (80 factorial) were used for the screening. For *Rf*CohScaB3, 1.0 μL drops of 22 and 47 mg·mL^−1^ of protein were mixed with 1.0 μL of reservoir solution at room temperature per well containing 50 μL of the crystallization solution. The same procedure was used for *Rf*CohScaA-Doc1b and *Rf*CohScaB3-Doc1a with protein drops at concentrations of 40 and 20 mg·mL^−1^ and 27 and 13.5 mg·mL^−1^, respectively. The resulting plates were then stored at 292 K.

Crystal formation from the initial screens was observed in the following 2 different conditions with the C-terminal tagged native *Rf*CohScaB3: 0.2 M lithium sulfate, 0.1 M sodium acetate pH 4.5, 30% w/v PEG 8000; and 0.17 M ammonium sulfate, 25.5% w/v PEG 4000, 15% v/v glycerol. SeMet-derivative plates were immediately set up for structure determination, should molecular replacement methods fail. For the SeMet-*Rf*CohScaB3 an optimization screen was set up around the range 0.1–0.5 M lithium sulfate, 0.1 M sodium acetate pH 4.5, 10–32% w/v PEG 8000 for the first condition; and 0.1–0.5 M ammonium sulfate, 10–32% w/v PEG 4000, 15% v/v glycerol for the second. The glycerol concentration was maintained at 15%, which acted as a cryoprotectant. Diffracting crystals were obtained in 12 of the 96 wells of the second optimization screen. These crystals grew to a maximum dimension of ~500 × 80 × 80^3^ μm, within two weeks. In addition, diffracting N-tagged *Rf*CohScaB3-Doc1a crystals were obtained in a 0.2 M ammonium nitrate and 20% w/v PEG 3550 solution while *Rf*ScaSCoh-Doc1b crystalized in a 0.2 M calcium acetate, 0.1 M sodium cacodylate trihydrate pH 6.5 and 18% PEG 8000 solution. All crystals were cryoprotected with mother solution containing 15–30% glycerol or with 100% Paratone-N (Hampton Research, USA) and flash-cooled in liquid nitrogen.

### Data collection, processing, structure determination and refinement

Data for the SeMet *Rf*CohScaB3 derivatives were collected on beamline ID23-2 at the European Synchrotron Radiation Facility (ESRF), Grenoble, France. 360° of data were collected with a ∆φ of 0.1° and an exposure of 0.04 sec. The data were collected at the wavelength of 0.8726 Å for a single-wavelength anomalous diffraction experiment. The crystal was cooled to 100 K using a gaseous nitrogen cryostream (Oxford Cryosystems) and data collected using the CCD MARMOSAIC 225 detector. The data sets were processed using iMOSFLM^28^ or XDS^29^ and AIMLESS^30^ from the CCP4 suite (Collaborative Computational Project, Number 4, 1994^31^). Data collection statistics are given in Table S1. The crystals belong to the tetragonal space group (*P*4_1_2_1_2), with a single molecule in the asymmetric unit, a solvent content of ~51% and a Matthews coefficient of ~2.49 Å^3^ Da^−1^ ^32^. The SeMet-*Rf*CohScaB3 structure was determined by single wavelength anomalous dispersion experiment with AUTOSOL^33^ from the PHENIX suite^34^). AUTOBUILD was used for building the initial structure^35^. Refmac5^36^ interspersed with model adjustment in COOT^37^ were used for structure refinement and rebuilding. PDB_REDO was used in the penultimate round of refinement for validation purposes^38^. The root mean square deviation of bond lengths, bond angles, torsion angles and other indicators were continuously monitored using validation tools in COOT and MOLPROBITY. Final coordinates and structure factors were deposited in PDB under accession codes 5AOZ and R5AOZSF, respectively.

Data for the Coh-Doc complexes were collected on beamline I04-1 at the Diamond Light Source, Harwell, England (*Rf*CohScaB3-Doc1a) and at the ESRF beamline ID-23, Grenoble, France (*Rf*CohScaA-Doc1b) using a PILATUS 6 M detector (Dectris Ltd). Data collection and processing was done as described above. Data collection statistics are given in Table S1. The best diffracting *Rf*CohScaB3-Doc1a crystals diffracted to a resolution of 1.26 Å and belonged to the orthorhombic space group *P*2_1_2_1_2_1_ with a single cohesin-dockerin complex in the asymmetric unit, a solvent content of ~43% and a Matthews coefficient of ~2.15 Å^3^ Da^−1^. PHASER^39^ was used to carry out molecular replacement using *Rf*CohScaB3 (5AOZ) and BUCCANEER^40^ helped building the initial dockerin model. Refinement and model rebuilding were carried out as described for *Rf*CohScaB3. The final round of refinement was performed using the TLS/restrained refinement procedure using each module as a single group. The best diffracting *Rf*CohScaA-Doc1b crystals diffracted to 1.70 Å and belonged to the orthorhombic spacegroup P2_1_2_1_2_1_ with a single cohesin-dockerin complex in the asymmetric unit, a solvent content of ~47% and a Matthews coefficient of ~2.33 Å^3^ Da^−1^. PHASER was used to carry out molecular replacement using the *Rf*CohScaB3-Doc1a model. Refinement occurred has described for *Rf*CohScaB3-Doc1a. A summary of the refinement statistics is shown in Table S1. Molecular representation figures were prepared with UCSF Chimera^19^. Final coordinates and structure factors were deposited in PDB under accession codes 5M2O and SF5M2O for *Rf*CohScaB3-Doc1a, and 5M2S and SF5M2S for *Rf*CohScaA-Doc1b, respectively.

### Data deposition

Coordinates and observed structure factor amplitudes have been deposited in the Protein Data Bank with the wwPDB entry codes 5AOZ (*Rf*CohScaB3), 5M2O (*Rf*CohScaB3-Doc1a) and 5M2S (*Rf*CohScaA-Doc1b).

## Electronic supplementary material

Supplementary information
