# Supplementary material for: Assembly of Ruminococcus flavefaciens cellulosome revealed by structures of two cohesin-dockerin complexes
Source: Sci Rep. 2017 Apr 7;7:759. doi: 10.1038/s41598-017-00919-w (PMC5429695; doi:10.1038/s41598-017-00919-w)
Supplement: Supplementary file 1 — Supplementary information [file 41598_2017_919_MOESM1_ESM.pdf]

SUPPLEMENTARY INFORMATION

Assembly of *Ruminococcus flavefaciens* cellulosome revealed by structures of two cohesin-dockerin complexes

Pedro Bule[a], Victor D. Alves[a], Vered Israeli-Ruimy[b], Ana Luísa Carvalho[c], Luís M.A. Ferreira[a], Steven P. Smith[d], Harry J. Gilbert[e], Shabir Najmudin[a], Edward A. Bayer[b] and Carlos M.G.A. Fontes[a,1]

[a] *CIISA – Faculdade de Medicina Veterinária, ULisboa, Pólo Universitário do Alto da Ajuda, Avenida da Universidade Técnica, 1300-477 Lisboa, Portugal*;

[b] *Department of Biomolecular Sciences, The Weizmann Institute of Science, Rehovot 76100 Israel;*

[c] *UCIBIO-REQUIMTE, Departamento de Química, Faculdade de Ciências e Tecnologia, Universidade Nova de Lisboa, 2829-516 Caparica, Portugal.*

[d] *Department of Biomedical and Molecular Sciences, Queen's University, Kingston, ON K7L 3N6, Canada*;

[e] *Institute for Cell and Molecular Biosciences, Newcastle University, The Medical School, Newcastle upon Tyne NE2 4HH, United Kingdom.*

Running title: Structure of *Ruminococcus flavefaciens* group 1 cohesin-dockerin complexes

[1] Corresponding author

E-mail: cafontes@fmv.ulisboa.pt

Keywords: Cellulosome, protein-protein interaction, protein structure, cellulose, cellulase, cohesin, dockerin.

Data deposition: Coordinates and observed structure factor amplitudes have been deposited in the Protein Data Bank with the wwPDB entry codes 5AOZ (*Rf*CohScaB3), 5M2O (*Rf*CohScaB3-Doc1a) and 5M2S (*Rf*CohScaA-Doc1b).

Table 1. X-ray crystallography data collection and refinement statistics for *Rf*CohScaB3, *Rf*CohScaB3-Doc1a and *Rf*CohScaA-Doc1b. Values in parenthesis are for the highest resolution shell.

**Dataset**	***Rf*CohScaB3**	***Rf*CohScaB3-Doc1a**	***Rf*CohScaA-Doc1b**
**Data Colection**			
Beamline	*ESRF ID23-2*	*Diamond I04-1*	*ESRF-ID23*
Space Group	$P4_12_12$	$P2_12_12_1$	$P12_11$
Wavelength (Å)	*0.8726*	*0.920*	*0.873*
Unit-cell parameters			
a, b c (Å)	*60.427, 60.427, 86.509*	*42.77, 63.51, 84.48*	*45.61, 64.49, 47.67*
α, β , γ (º)	*90, 90, 90*	*90, 90, 90*	*90, 116.72, 90*
V_m*# (Å3 Da-1)*	*2.36*	*2.15*	*2.33*
Solvent Content (%)	*48.01*	*42.94*	*47.27*
Resolution limits (Å)	*49.54 – 1.14 (1.18 – 1.14)*	*20.27 – 1.26 (1.305– 1.26)*	*42.58 – 1.7 (1.761 – 1.7)*
No. of observations	*606740 (55700)*	*460418 (38386)*	*112328 (7417)*
No. of unique observations	*58923 (5791)*	*62519 (6069)*	*26481 (2322)*
Multiplicity	*10.3 (9.6)*	*7.4 (6.3)*	*4.2 (3.2)*
Completeness (%)	*99.91 (99.27)*	*99.6 (98.09)*	*97.38 (86.13)*
<I/σ(I)>	*18.21 (1.73)*	*5.74 (2.56)*	*9.33 (4.34)*
CC1/2†	*0.999 (0.582)*	*0.976 (0.783)*	*0.991 (0.845)*
Wilson B-factor	*11.66*	*6.76*	*8.48*
Rmerge ‡	*0.073 (1.327)*	*0.2322 (0.5811)*	*0.1134 (0.2575)*
Strtucture Refinement			
R-work § , R-free ¥	*0.1184, 0.1424*	*0.1318, 0.1535*	*0.1313, 0.1592*
No. of Non-H atoms	*1331*	*1947*	*2041*
Macromolecules	*1100*	*1622*	*1695*
Ligands	*6*	*2*	*21*
Water	*225*	*323*	*325*
Protein residues	*141*	*211*	*220*
RMS(bonds)	*0.016*	*0.0178*	*0.019*
RMS(angles)	*1.75*	*1.780*	*1.87*
Ramachandran favored (%)	*95*	*97.2*	*98*
Ramachandran outliers (%)	*0*	*0*	*0*
Clash score	*2.71*	*1.24*	*3.2*
Average B-factor	*17.60*	*10*	*12.50*
macromolecules	*14.80*	*7.5*	*9.80*
ligands	*16.30*	*4.4*	*25.60*
solvent	*31.00*	*22.90*	*25.70*
PDB accession code	*5AOZ*	*5M2O*	*5M2S*

# V_m = Matthews coefficient.
† $CC_{1/2}$ = the correlation between intensities from random half-dataset.
‡ $R_{merge} = \Sigma_{hkl} \Sigma_i |I_i(hkl) - \langle I(hkl)\rangle| / \Sigma_{hkl} \Sigma_i I_i(hkl)$, where $I_i(hkl)$ is the *i*th intensity measurement of reflection *hkl*, including symmetry-related reflections and $\langle I(hkl)\rangle$ is its average.
§Rwork = $\Sigma_{hkl}||F_{obs}|-|F_{calc}||/\Sigma_{hkl}|F_{obs}|$.
¥R_{free} as R_{work}, but summed over a 5% test set of reflections.

TABLE S2. Main hydrophobic contacts between *Rf*CohScaB3 and *Rf*Doc1a and *Rf*CohScaA and *Rf*Doc1b. Table was made using the PDBePISA server. Some of the dockerin residues are marked as belonging either to helix 1 (H1) or to helix 3 (H3) interfaces.

	***Rf*Doc1a**			***Rf*CohScaB3**
	**Residue**	**Residue #**		**Residues**
	ASN	32	< >	ASN124, ASP125, GLY126
	ASP	34	< >	ASP125, GLY126
	ASP	38	< >	HIS121
H1	ILE	39	< >	ALA38, MET39, PHE76, HIS121
H1	SER	40	< >	HIS121, SER123, ASN 124, GLY126
H1	VAL	43	< >	SER37, ALA38, SER123
H1	MET	46	< >	LYS77, LEU79
H1	GLN	47	< >	GLY83, ASN124
H1	ALA	50	< >	ASP81, LYS82, GLY83
	ASN	51	< >	LYS82
	LYS	54	< >	GLU84
	TYR	55	< >	GLU84, ASN124
H3	GLN	80	< >	MET66
H3	GLN	83	< >	MET66, ASN75, PHE76, LYS77
H3	SER	84	< >	MET66
H3	CYS	86	< >	ASP40, ASN75, LYS117
H3	LEU	87	< >	MET66, ASN68, ILE71, GLY73, ALA74, ASN75
	LEU	89	< >	ASN68
	***Rf*Doc1b**			***Rf*CohScaA**
	**Residue**	**Residue #**		**Residues**
	ASN	32	< >	ASN124, ASP125, GLY126
	ASP	34	< >	ASP125, GLY126
	ASP	38	< >	HIS121
H1	ILE	39	< >	ALA39, PHE77, HIS121,
H1	SER	40	< >	HIS121, ASN124, GLY126, SER 123, ASP125
H1	VAL	43	< >	SER123, ASN124, SER38, ALA39
H1	ILE	44	< >	ASN124
H1	MET	46	< >	LEU80, LYS 78,
H1	GLN	47	< >	GLY84, ASN124
H1	SER	50	< >	ASP82, LYS83, GLY84
H1	ASN	51	< >	LYS83
	LYS	54	< >	GLU85
	PHE	55	< >	GLU85
	HIS	63	< >	ASN124
H3	LEU	88	< >	MET67
H3	GLN	91	< >	ASN76, MET67, LYS78
H3	LYS	92	< >	MET67
H3	LEU	94	< >	LYS117, ASP41, ASN76
H3	LEU	95	< >	ILE72, ASN69, ASN76, MET67, GLY74, ALA75
H3	ASN	96	< >	ASN69
H3	LEU	97	< >	THR68, ASN69

TABLE S3. Recombinant protein sequences of *Rf*Doc1a, *Rf*Doc1b, *Rf*CohScaA, *Rf*CohScaB3 and mutant variants of these proteins produced for the interaction studies. The mutated residues are highlighted in black.

**Dockerin**	**Protein Sequence**
**Doc1a**	EAVQKFPGDANCDGIVDISDAVLIMQTMANPSKYQMTDKGRINADVTGNSDGVTVLDAQFIQSYCLGLVELPPVEYVNVTKQPVEPA
**Doc1a I39A**	EAVQKFPGDANCDGIVDASDAVLIMQTMANPSKYQMTDKGRINADVTGNSDGVTVLDAQFIQSYCLGLVELPPVEYVNVTKQPVEPA
**Doc1a S40A**	EAVQKFPGDANCDGIVDIADAVLIMQTMANPSKYQMTDKGRINADVTGNSDGVTVLDAQFIQSYCLGLVELPPVEYVNVTKQPVEPA
**Doc1a V43A**	EAVQKFPGDANCDGIVDISDAALIMQTMANPSKYQMTDKGRINADVTGNSDGVTVLDAQFIQSYCLGLVELPPVEYVNVTKQPVEPA
**Doc1a Q47A**	EAVQKFPGDANCDGIVDISDAVLIMATMANPSKYQMTDKGRINADVTGNSDGVTVLDAQFIQSYCLGLVELPPVEYVNVTKQPVEPA
**Doc1a K54A**	EAVQKFPGDANCDGIVDISDAVLIMQTMANPSAYQMTDKGRINADVTGNSDGVTVLDAQFIQSYCLGLVELPPVEYVNVTKQPVEPA
**Doc1a Q83A**	EAVQKFPGDANCDGIVDISDAVLIMQTMANPSKYQMTDKGRINADVTGNSDGVTVLDAQFIASYCLGLVELPPVEYVNVTKQPVEPA
**Doc1a L87A**	EAVQKFPGDANCDGIVDISDAVLIMQTMANPSKYQMTDKGRINADVTGNSDGVTVLDAQFIQSYCAGLVELPPVEYVNVTKQPVEPA
**Doc1a I39A + V43A**	EAVQKFPGDANCDGIVDASDAALIMQTMANPSKYQMTDKGRINADVTGNSDGVTVLDAQFIQSYCLGLVELPPVEYVNVTKQPVEPA
**Doc1a V43A + Q47A**	EAVQKFPGDANCDGIVDISDAALIMATMANPSKYQMTDKGRINADVTGNSDGVTVLDAQFIQSYCLGLVELPPVEYVNVTKQPVEPA
**Doc1b**	NVTLWGDANCDGIVDISDAVIIMQSLSNPSKFDRNGNDEHHITAQGELNGDVNENGNGITNADALAIQKYLLNLIGNLPE
**Cohesin**	**Protein Sequence**
**CohScaB3 WT**	MPVANADVVFDFQNYTAKAGDEVTVDVLVDSKNK PISAMDVKFKVDSPLTIEEIDKESLAFNTTVMTNMAILGANFKSLDDKGEPLVPKDGAAVFTLYVNVPANTPDGTYYVGFNGKNEVHKSNDGS QFTVASKNGAITVGTPNEEG
**CohScaB3 A38Q**	...PISQMDVKFKVDSPLTIEEIDKESLAFNTTVMTNMAILGANFKSLDDKGEPLVPKDGAAVFTLYVNVPANTPDGTYYVGFNGKNEVHKSNDGS...
**CohScaB3 N68A**	...PISAMDVKFKVDSPLTIEEIDKESLAFNTTVMTAMAILGANFKSLDDKGEPLVPKDGAAVFTLYVNVPANTPDGTYYVGFNGKNEVHKSNDGS...
**CohScaB3 N75A**	...PISAMDVKFKVDSPLTIEEIDKESLAFNTTVMTNMAILGAAFKSLDDKGEPLVPKDGAAVFTLYVNVPANTPDGTYYVGFNGKNEVHKSNDGS...
**CohScaB3 K77A**	...PISAMDVKFKVDSPLTIEEIDKESLAFNTTVMTNMAILGANFASLDDKGEPLVPKDGAAVFTLYVNVPANTPDGTYYVGFNGKNEVHKSNDGS...
**CohScaB3 L79A**	...PISAMDVKFKVDSPLTIEEIDKESLAFNTTVMTNMAILGANFKSADDKGEPLVPKDGAAVFTLYVNVPANTPDGTYYVGFNGKNEVHKSNDGS...
**CohScaB3 E84A**	...PISAMDVKFKVDSPLTIEEIDKESLAFNTTVMTNMAILGANFKSLDDKGAPLVPKDGAAVFTLYVNVPANTPDGTYYVGFNGKNEVHKSNDGS...
**CohScaB3 H121A**	...PISAMDVKFKVDSPLTIEEIDKESLAFNTTVMTNMAILGANFKSLDDKGEPLVPKDGAAVFTLYVNVPANTPDGTYYVGFNGKNEVAKSNDGS...
**CohScaB3 N124A**	...PISAMDVKFKVDSPLTIEEIDKESLAFNTTVMTNMAILGANFKSLDDKGEPLVPKDGAAVFTLYVNVPANTPDGTYYVGFNGKNEVHKSADGS...
**CohScaB3 N75A + E84A**	...PISAMDVKFKVDSPLTIEEIDKESLAFNTTVMTNMAILGAAFKSLDDKGAPLVPKDGAAVFTLYVNVPANTPDGTYYVGFNGKNEVHKSNDGS...
**CohScaB3 N75A + H121A**	...PISAMDVKFKVDSPLTIEEIDKESLAFNTTVMTNMAILGAAFKSLDDKGEPLVPKDGAAVFTLYVNVPANTPDGTYYVGFNGKNEVAKSNDGS...
**CohScaB3 N75A + N124A**	...PISAMDVKFKVDSPLTIEEIDKESLAFNTTVMTNMAILGAAFKSLDDKGEPLVPKDGAAVFTLYVNVPANTPDGTYYVGFNGKNEVHKSADGS...
**CohScaB3 E84A + H121A**	...PISAMDVKFKVDSPLTIEEIDKESLAFNTTVMTNMAILGANFKSLDDKGAPLVPKDGAAVFTLYVNVPANTPDGTYYVGFNGKNEVAKSNDGS...
**CohScaB3 E84A + N124A**	...PISAMDVKFKVDSPLTIEEIDKESLAFNTTVMTNMAILGANFKSLDDKGAPLVPKDGAAVFTLYVNVPANTPDGTYYVGFNGKNEVHKSADGS...
**CohScaB3 H121A + N124A**	...PISAMDVKFKVDSPLTIEEIDKESLAFNTTVMTNMAILGANFKSLDDKGEPLVPKDGAAVFTLYVNVPANTPDGTYYVGFNGKNEVAKSADGS...
**CohScaA**	MQPVANADVIFDFGNYEAKAGEEVQVDVTVDSKNKAISAMDVVFAIDSPLTIDEIDKESLAFKTTAMTNIAILGANFKSLDDKGEPLVPTKDPVFTLYV TVPATTPDGVYNVGFGNKCEVHKSNDGSKYSSTAINGKIKVGNPVDDP

TABLE S4. Primers used to isolate genes encoding *R. flavefaciens* dockerins *Rf*Doc1a and *Rf*Doc1b and to generate the Doc1a and CohScaB3 mutant derivatives. Sequences used for plasmid recombination are in italic.

Dockerin	Vector	Primers used
Doc1a	pHTP2	5’ *TCAGCAAGGGCTGAGG*GTTCAGAAGTTCCCG<br>3’ *TCAGCGGAAGCTGAGGTTA*TTCAACAGGCGGAAG
Doc1b	pHTP2	5’ *TCAGCAAGGGCTGAGG*AATGTTACTCTCTGG<br>3’ *TCAGCGGAAGCTGAGGTTA*CTCTGGAAGATTTCC
CohScaB3 A38Q	pET28a	5’ GAACAAGCCAATCTCACAGATGGACGTTAAGTTC<br>3‘ GAACTTAACGTCCATCTGTGAGATTGGCTTGTTC
CohScaB3 N68A	pET28a	5’ CAACAGTCATGACAGCCATGGCTATCCTTGG<br>3‘ CCAAGGATAGCCATGGCTGTCATGACTGTTG
CohScaB3 N75A	pET28a	5’ GCTATCCTTGGTGCAGCCTTCAAGTCACTCGAC<br>3‘ GTCGAGTGACTTGAAGGCTGCACCAAGGATAGC
CohScaB3 K77A	pET28a	5’ CTTGGTGCAAACTTCGCGTCACTCGACGATAAG<br>3‘ CTTATCGTCGAGTGACGCGAAGTTTGCACCAAG
CohScaB3 L79A	pET28a	5’ GCAAACTTCAAGTCAGCCGACGATAAGGGCGAAC<br>3‘ GTTCGCCCTTATCGTCGGCTGACTTGAAGTTTGC
CohScaB3 E84A	pET28a	5’ CTCGACGATAAGGGCGCACCGCTCGTTCCTAAG<br>3‘ CTTAGGAACGAGCGGTGCGCCCTTATCGTCGAG
CohScaB3 H121A	pET28a	5’ GGAAAGAACGAAGTAGCCAAGAGCAACGACGG<br>3‘ CCGTCGTTGCTCTTGGCTACTTCGTTCTTTCC
CohScaB3 N124A	pET28a	5’ GAAGTACACAAGAGCGCCGACGGTTCACAGTTC<br>3‘ GAACTGTGAACCGTCGGCGCTCTTGTGTACTTC
Doc1a I39A	pHTP2	5’ GACGGAATAGTTGATGCTTCGGATGCAGTACTC<br>3‘ GAGTACTGCATCCGAAGCATCAACTATTCCGTC
Doc1a S40A	pHTP2	5’ GGAATAGTTGATATTGCGGATGCAGTACTC<br>3‘ GAGTACTGCATCCGCAATATCAACTATTCC
Doc1a V43A	pHTP2	5’ GATATTTCGGATGCAGCACTCATTATGCAGAC<br>3‘ GTCTGCATAATGAGTGCTGCATCCGAAATATC
Doc1a Q47A	pHTP2	5’ GCAGTACTCATTATGGCGACTATGGCTAATCC<br>3‘ GGATTAGCCATAGTCGCCATAATGAGTACTGC
Doc1a K54A	pHTP2	5’ GGCTAATCCAAGCGCATATCAGATGACCGAC<br>3‘ GTCGGTCATCTGATATGCGCTTGGATTAGCC
Doc1a Q83A	pHTP2	5’ GATGCACAGTTCATAGCGAGCTATTGTCTGGGA<br>3‘ TCCCAGACAATAGCTCGCTATGAACTGTGCATC
Doc1a L87A	pHTP2	5’ CATACAGAGCTATTGTGCGGGACTTGTTGAACTTC<br>3‘ GAAGTTCAACAAGTCCCGCACAATAGCTCTGTATG

TABLE S5. Primers used to amplify the cohesins and group1 Docs used in the cellulose microarray assays.

Dockerin	Vector	Primers used
Doc1132_a	pET9d	5’ gctacggtacct GAG CGT GTT ACT CTG TGG<br>3’ cgccagggatcc TTA TCA GTT ATA GCT CTC GGG
Doc1222_a	pET9d	5’ gctacggtacct GTA ACA CTC TGG GGC GAT GCT<br>3’ cgccagggatcc TTA TGC GAT ATA TGT CTT ATT TGA TGC
Doc1315_a	pET9d	5’ gctacggtacct ACA CTC TGG GGC GAT GCC<br>3’ cgccagggatcc TTA CTG ATA ATT TGA TCT TGA GGC
Doc1_a	pET9d	5’ gctacggtacct GAG GCT GTT CAG AAG TTC<br>3’ cgccagggatcc TTA TTC GGG CTC ATA GTA AAC
DocScaO_a	pET9d	5’ gctac ggtacct TCT GTA ACT TCA ACA GTC AAA G<br>3’ cgccagggatcc TTAACT CTC CAC AAA CTC CCA GT
Doc3925_a	pET9d	5’ gctacggtacct GTT CTC TGG GGC GAT GCT<br>3’ cgccagggatcc TTA TGA CTC AGG GAG CTT AGT
DocScaM_a	pET9d	5’ gctac ggtacct TTA GAG ATA GTT CTT GAT GAA CC<br>3’ cgccagggatcc TTA ATC AAG CTT CAG CAG TTT TTT C
Doc1327_b	pET9d	5’ gctacggtacct GCT ACT ATC GTT GGT GAC<br>3’ cgccagggatcc TTA TTA CTT AGT TGT TGG GAG AG
Doc4293_b	pET9d	5’ gctacggtacct GGA CTT GCA GGC GAT ACC<br>3’ cgccagggatcc TTA TCA GCT TGT CAG CTT GTC
DocScaC_b	pET9d	5’ gctacggtacct CCC GAT CAG GCT ACT CTG<br>3’ cgccagggatcc TTA TCA AAG TTC TGT GAT GAG AG
Doc0535_c	pET9d	5’ gctacggtacct GCC GGT ATT CTC TGG GGC<br>3’ cgccagggatcc TTA TTA TTT GCT ATA GGA TTC GGG
DocScaJ_d	pET9d	5’ gctacggtacct ACT GCT GCT GAG CCT GTA<br>3’gccagggatcc TTA ATG TCA TTA TTC AAG CTT CAG
**Cohesin**	**Vector**	**Primers used**
CohScaA	pET28a	5’ gtccatggatcc CAG ACA AGT GGT ACT CCT TCC<br>3’ cagcttctcgag TTA AGC TGT TGT AGC AGA TGT TGT TGG ATC
CohScaB2	pET28a	5’ gtccatggatcc CAG ACA AGT GGT ACT CCT TCC<br>3’ cagcttctcgag TTA TGA GCC TGA ACC TGT TGT AGG
CohScaB6	pET28a	5’ gtccatggatcc ACT GAT ACA AAC GGT AAC AAG<br>3’ cagcttctcgag TTA TGT AAG AGT GAT CTT ATC AGT
CohScaC	pET28a	5’ gtccatggatcc GCT CCG GCA TTC GCT GCA<br>3’ cagcttctcgag TTA AGC CTT GGT GGT TGT TAC TTC
CohScaE	pET28a	5’ actaccatgg CGCTCACAGACAGAGGAATG<br>3’ actactcgag TGGCTCACCAGCCTTGATTGC
CohScaF	pET28a	5’ gtccatggatcc AAT TCA ACA GAT CTC ACC GAA GC<br>3’ cagcttctcgag TTA GCC AAG CTT ATA CTC AGT AG
CohScaG	pET28a	5’ gtccatggatcc AGC GGC GGA AGC AGT TCG<br>3’ cagcttctcgag TTATTC AAC TGT TAT AGT GCC GCC
CohScaH	pET28a	5’ gtccatggatcc GCC TGC CCA GAT CGT GGA<br>3’ cagcttctcgag TTA CGT TTC GGA AGG AGC GGT
CohScaI	pET28a	5’ gtccatggatcc GGC CCC GTA GTT CAG GGA AAG<br>3’ cagcttctcgag TTA ATC GGC AAC TAT CTC GAT GGC
CohScaJ1	pET28a	5’ gtccatggatcc GCT GAA ACA TCA ACA GCA<br>3’ cagcttctcgag TTA AGA AGT TTC GGT TGT AAC
CohScaJ2	pET28a	5’ gtccatggatcc TCT ACA AAA ACA AAC ACC CAA ACA<br>3’ cagcttctcgag TTA AGC AGC AGT AGT TGT TGT TAT TAC
CohScaO	pET28a	5’ gtccatggatcc GCG CCT GTT ACA ATA TCA G<br>3’ cagcttctcgag TTAAGT AGT ACT TAC CTG AGA A

SUPPLEMENTARY FIGURE LEGENDS

FIGURE S1. Structure of *Rf*CohScaB3. *A.* The structure of CohScaB3 is represented in color ramped style from the blue N-terminus to the red C-terminus. Below the transparent molecular surface, the most important residues for dockerin interaction are shown in ball&stick representation, above the pink oval disk that marks the plane defined by the 8-3-6-5 β-sheets. Each of the 9 β-strands is labeled. *B.* Overlay of *Rf*CohScaB3 with *Rf*CohScaC, with the blue and tan colored transparent molecular surface, respectively, revealing the secondary structure and the major differences, particularly at the dockerin-interacting plateau highlighted above the same oval pink plane representation. *Rf*CohScaB3 cyan-colored residues N-68 and N-124 were left on panel B. as orientation reference points relative to panel A.

FIGURE S2. Topology diagram of CohScaB3 compared with previously described cohesins and *Rf*CohScaC. The CohScaB3 module (last) forms the classical nine-stranded β-sandwich with jelly-roll topology, which is essentially analogous to that of the cohesin modules *Ac*ScaCCoh3 (first, PDB code 4UYP) and *Bc*ScaACoh11 (second, PDB code 1TYJ), respectively. Unlike *Ac*ScaCCoh3 and *Bc*ScaACoh11, *Rf*CohScaC (third, PDB code 5LXV) does not possess any β-flap extensions interrupting β-strands or α-helices between β-strands.

FIGURE S3. Dockerin *Rf*Doc1a calcium octahedral coordination. The left and right panels show a representation of the *Rf*Doc1a N- and C-terminal Ca^{2+} ions, respectively. In both panels the secondary structure ribbon representation of *Rf*Doc1a highlights the amino-acid residues (in stick representation) involved in the metal coordination, surrounded by a transparent light yellow representation of the Refmac5 maximum-likelihood σ_A–weighted $2F_o{-}F_c$ electron density map contoured at 1σ (0.46 electrons/A^3). The labels show the *Rf*Doc1a residue and coordination position numbers and also the atoms involved. Both calcium ions are depicted as purple spheres and are overlaid with an idealized octahedral geometry representation (green arrows). A single water molecule (Wat) completes the coordination sphere. The bidentate nature of the Asp-34 and Asp-78 coordination is highlighted with blue dashed lines.

FIGURE S4. Electrostatic surface potential for the Coh-Doc interface. In each panel the right images show the cohesin binding plateau with the bound dockerin partner in N- to C-terminus rainbow color-ramped style on top, while the left images depict the same complex after a 180º rotation along axis "X", thus allowing the view of the molecular dockerin-binding surface, below a transparent view of the secondary structure of the Coh partner. *A. Rf*CohScaB3 and *Rf*Doc1a (PDB code 5M2O). *B. Rf*CohScaA and *Rf*Doc1b (PDB code 5M2S). *C. Rf*CohScaC and *Rf*Doc3 (PDB code 5LXV) and *D. Ct*CohScaA2 and *Ct*DocXyn10B (PDB code 2CCL). The figure was prepared with UCSF Chimera using APBS (Adaptive Poisson-Boltzmann Solver) and the electrostatic potential was contoured from -6 (red) to +6 (blue) (arbitrary Chimera units).

FIGURE S5. Binding affinity of wild-type *Rf*Doc1a and 1b to both *Rf*CohScaB3 and *Rf*CohScaA determined by ITC. Binding isotherms for *A. Rf*CohScaB3 vs *Rf*Doc1a, *B. Rf*CohScaA vs *Rf*Doc1a, *C. Rf*CohScaB3 vs *Rf*Doc1b and *D. Rf*CohScaA vs *Rf*Doc1b are displayed. The upper part of each panel shows the raw heats of binding, whereas the lower parts comprise the integrated heats after correction for heat of dilution. The curve represents the best fit to a single-site binding model. The corresponding thermodynamic parameters are shown in Table 2.

FIGURE S6. Binding affinity of CohScaB3 and Doc1a mutant derivatives to their wild-type partners, determined by non-dentaturing gel electrophoresis (NGE). *A.* The lanes marked D5 were loaded with the dockerin alone. Adjacent lanes were loaded with the cohesin mutant derivatives and with both cohesin and dockerin modules after 60-min incubation at equimolar concentrations. *B.* The lanes marked ScaB3 were loaded with the cohesin alone. Adjacent lanes were loaded with the dockerin mutant derivatives and with both cohesin and dockerin modules after 60-min incubation at equimolar concentrations. The appearance of a band with a different migration pattern in lanes containing the complex represents a positive

result (e.g. ScaB3), while a negative result (e.g. ScaB3 A38Q) is given by the appearance of only the individual dockerin and cohesin bands.

FIGURE S7. Coh-binding range of *R. flavefaciens* group 1 dockerins. Each bar graph represents the recognition profile of one dockerin from a different group 1 subgroup and 12 cohesins. The bar values correspond to the ratio between the measured Cy3 and Cy5 signals. Intensity values were calculated by Array Vision Evaluation 8.0 software and all data processing was made in ExcelIntensity values were calculated by Array Vision Evaluation software and all data processing was made in Excel.

FIGURE S8. Multiple sequence alignment of *R. flavefaciens* ScaA, ScaB and ScaC cohesins. The primary sequence background is colored according to the ALSCRIPT Calcons convention, implemented in ALINE (39): red, identical residues; orange to blue, lowering color-ramped scale of conservation. Above and below the alignment lies a cartoon representation of the secondary structure of CohScaB3 (blue color) and CohScaC (purple color), respectively, with the β-strands numbering. Also for these two Cohs, the residues involved in molecular interactions with the Doc partner (Coh-Doc complexes PDB codes: 5m2o and 5lxv, respectively) are represented as follows: blue triangle for hydrogen bonds, red triangle for salt bridges and yellow circles for hydrophobic contacts. Critical residues for *Rf*CohScaB3/*Rf*CohScaA Doc-binding are marked with a black box and labelled on the top.

FIGURE S1

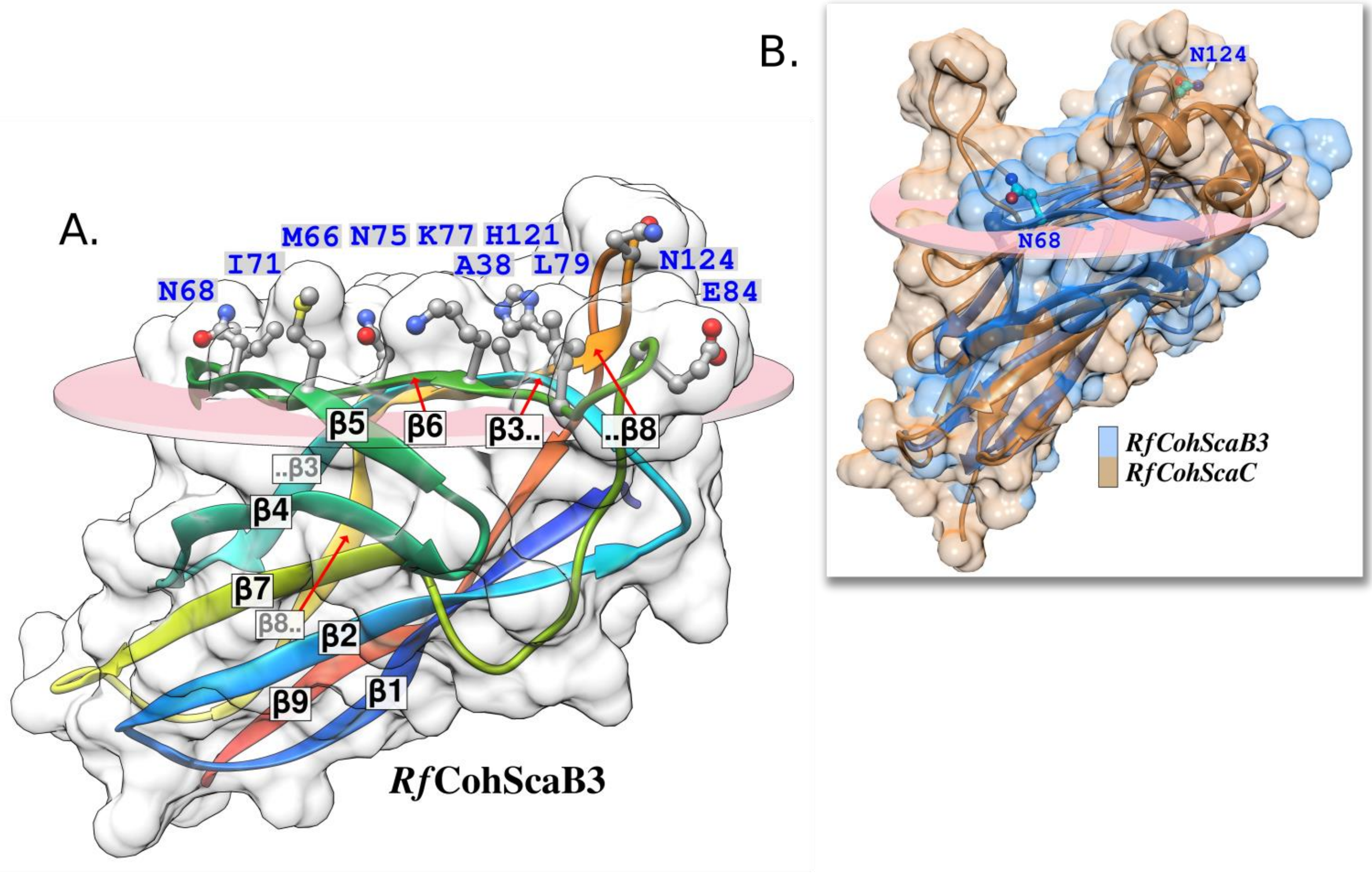

A.
N68
I71
M66
N75
K77
H121
A38
L79
N124
E84
β5
β6
β3..
..β8
..β3
β4
β7
β8..
β2
β9
β1
RfCohScaB3
B.
N124
N68
RfCohScaB3
RfCohScaC

FIGURE S2

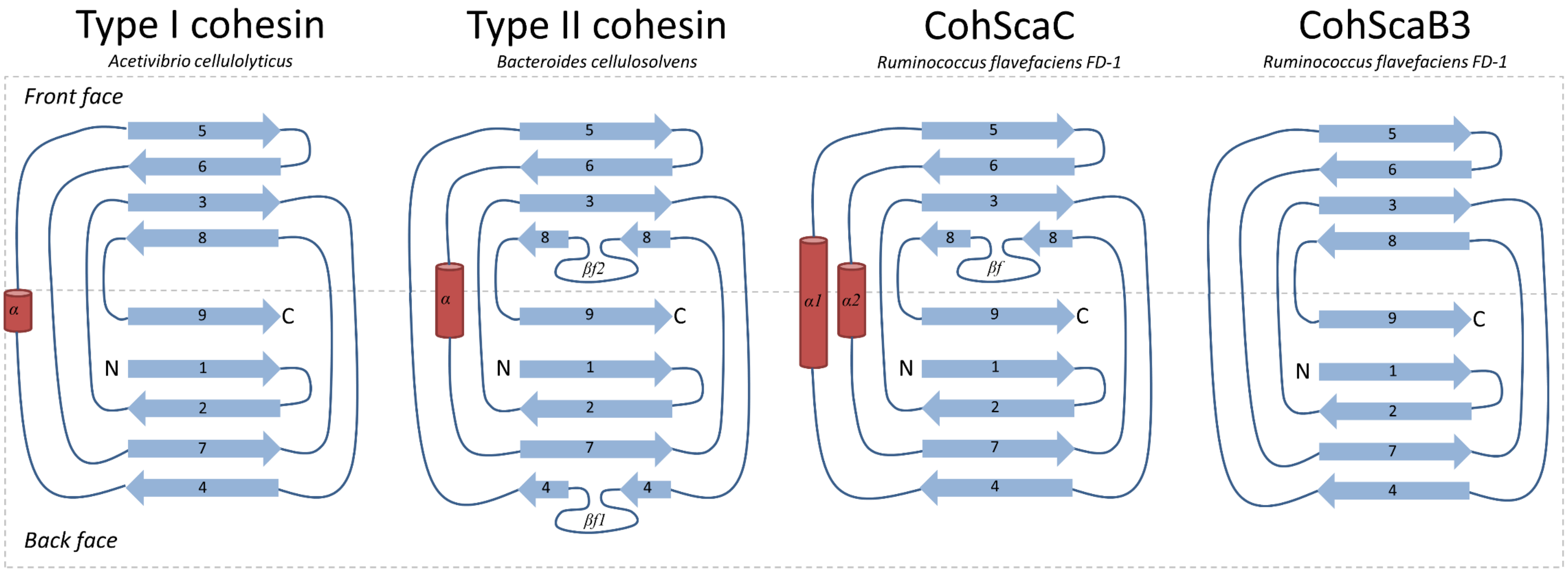

Type I cohesin
Acetivibrio cellulolyticus
Type II cohesin
Bacteroides cellulosolvens
CohScaC
Ruminococcus flavefaciens FD-1
CohScaB3
Ruminococcus flavefaciens FD-1
Front face
Back face
5
6
3
8
9
C
N
1
2
7
4
α
βf2
βf1
α1
α2
βf

FIGURE S3

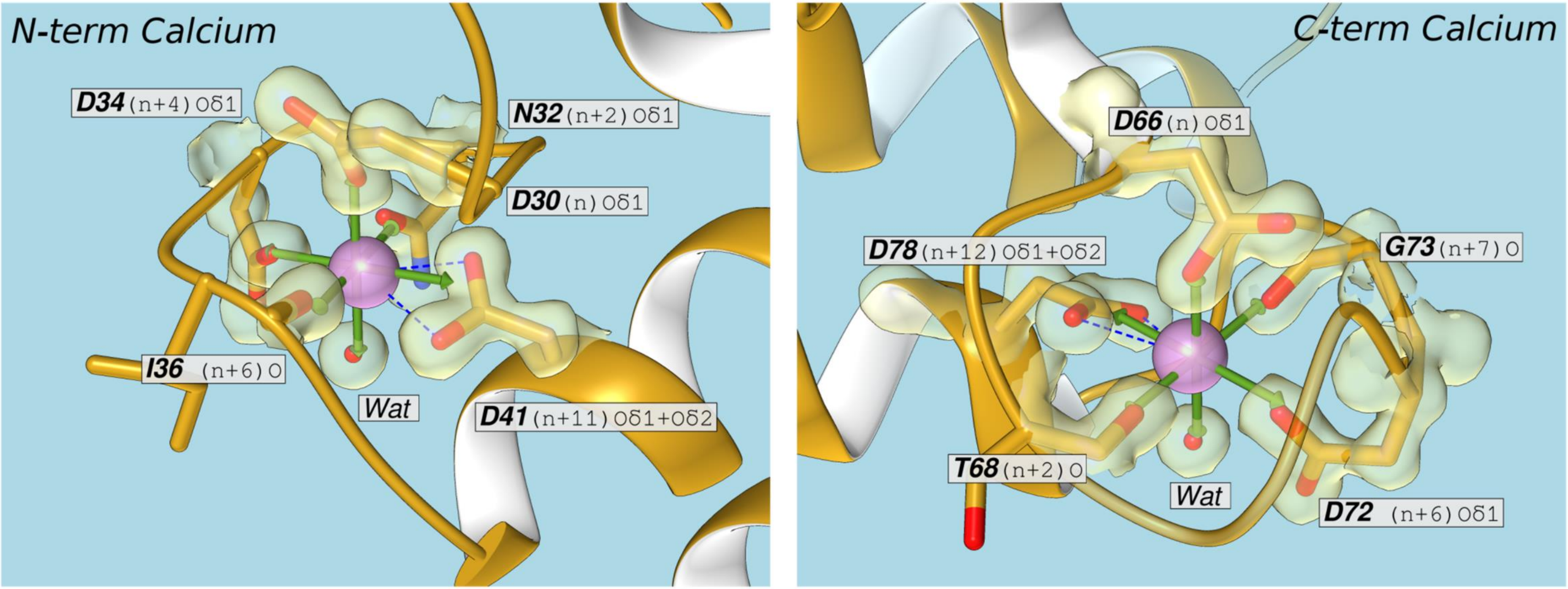

N-term Calcium
D34 (n+4) Oδ1
N32 (n+2) Oδ1
D30 (n) Oδ1
I36 (n+6) O
Wat
D41 (n+11) Oδ1+Oδ2
C-term Calcium
D66 (n) Oδ1
D78 (n+12) Oδ1+Oδ2
G73 (n+7) O
T68 (n+2) O
Wat
D72 (n+6) Oδ1

FIGURE S4

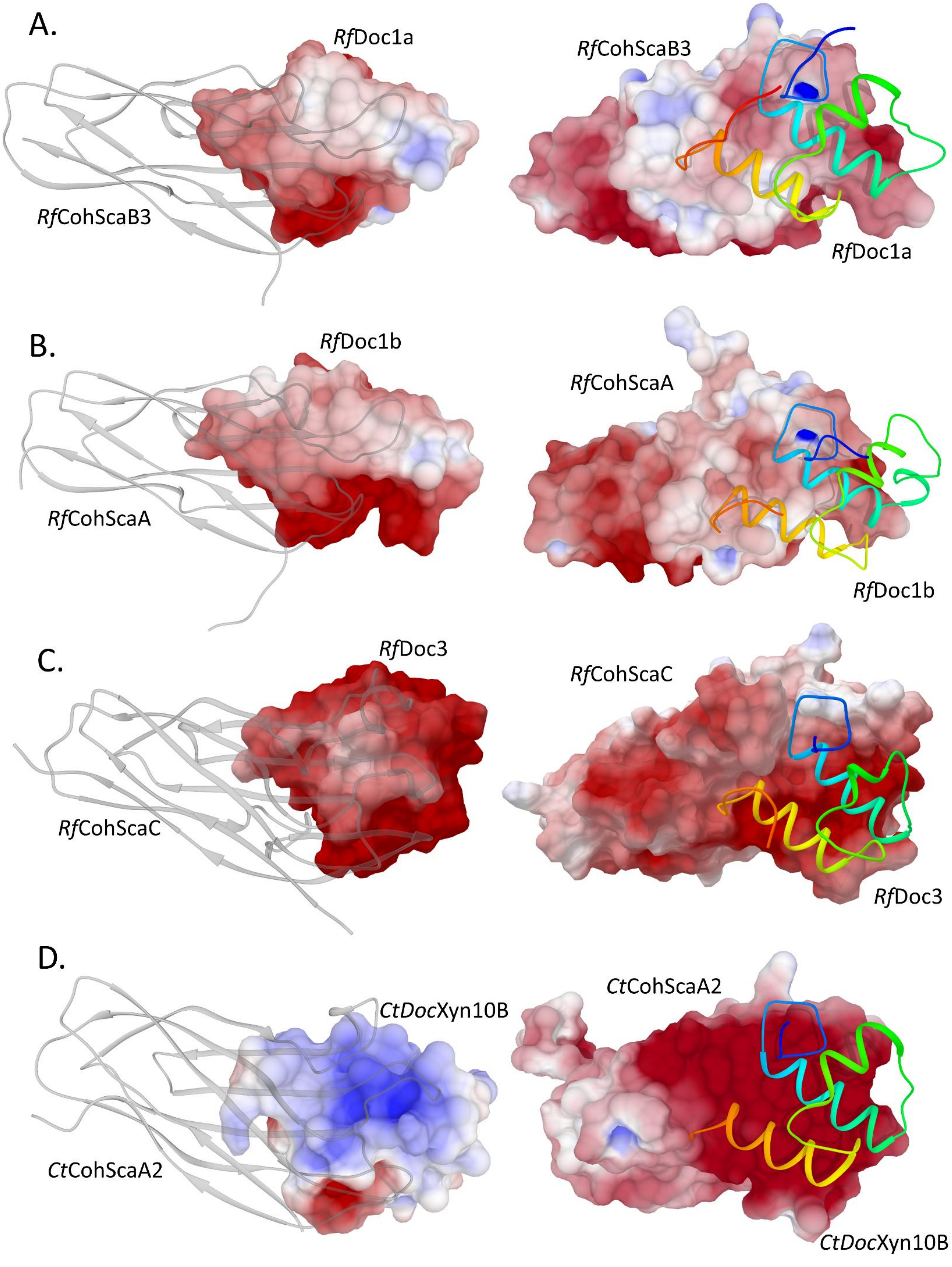
A.
RfDoc1a
RfCohScaB3
RfCohScaB3
RfDoc1a
B.
RfDoc1b
RfCohScaA
RfCohScaA
RfDoc1b
C.
RfDoc3
RfCohScaC
RfCohScaC
RfDoc3
D.
CtDocXyn10B
CtCohScaA2
CtCohScaA2
CtDocXyn10B

FIGURE S5

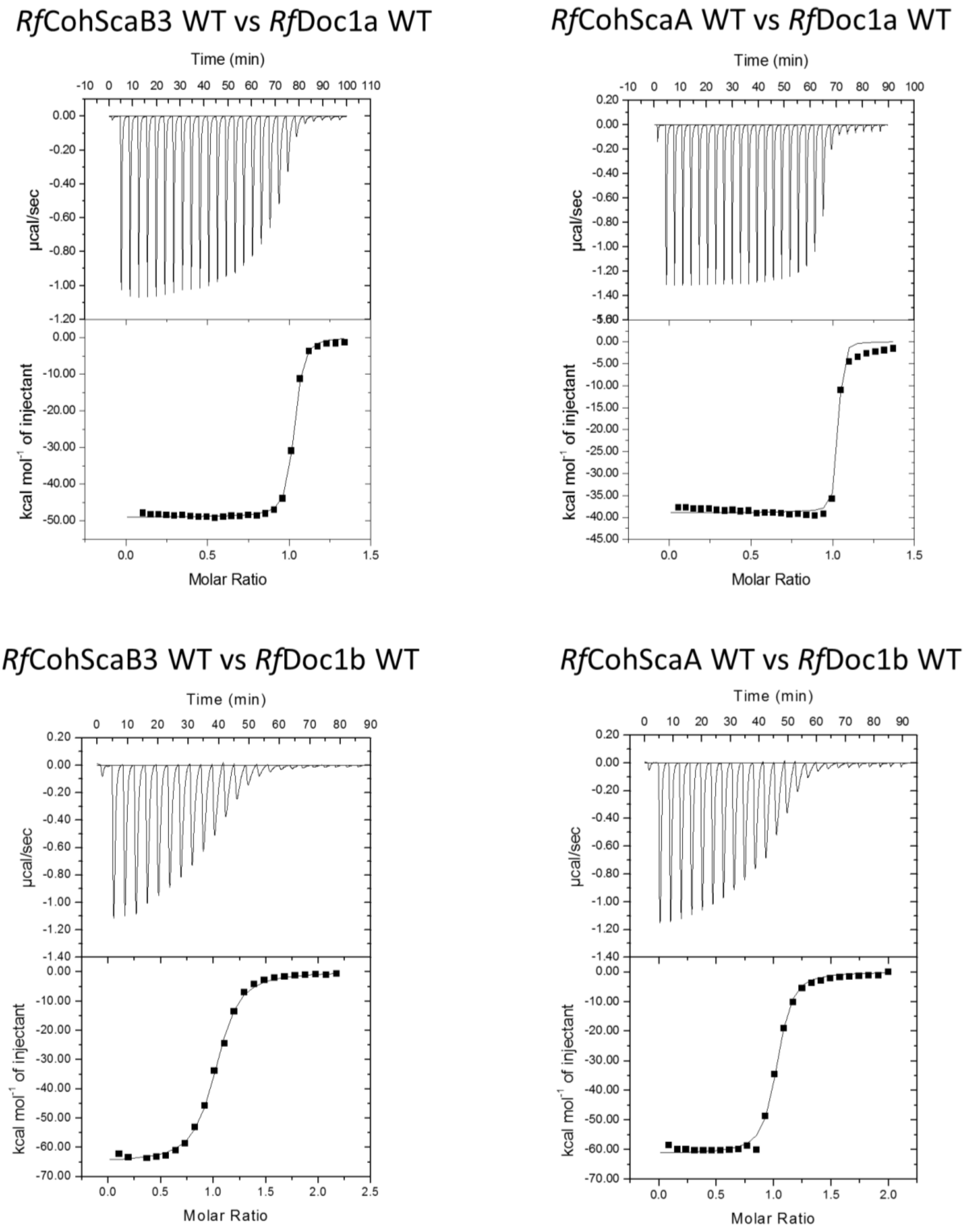

RfCohScaB3 WT vs RfDoc1a WT
RfCohScaA WT vs RfDoc1a WT
RfCohScaB3 WT vs RfDoc1b WT
RfCohScaA WT vs RfDoc1b WT
Time (min)
µcal/sec
kcal mol⁻¹ of injectant
Molar Ratio

FIGURE S6

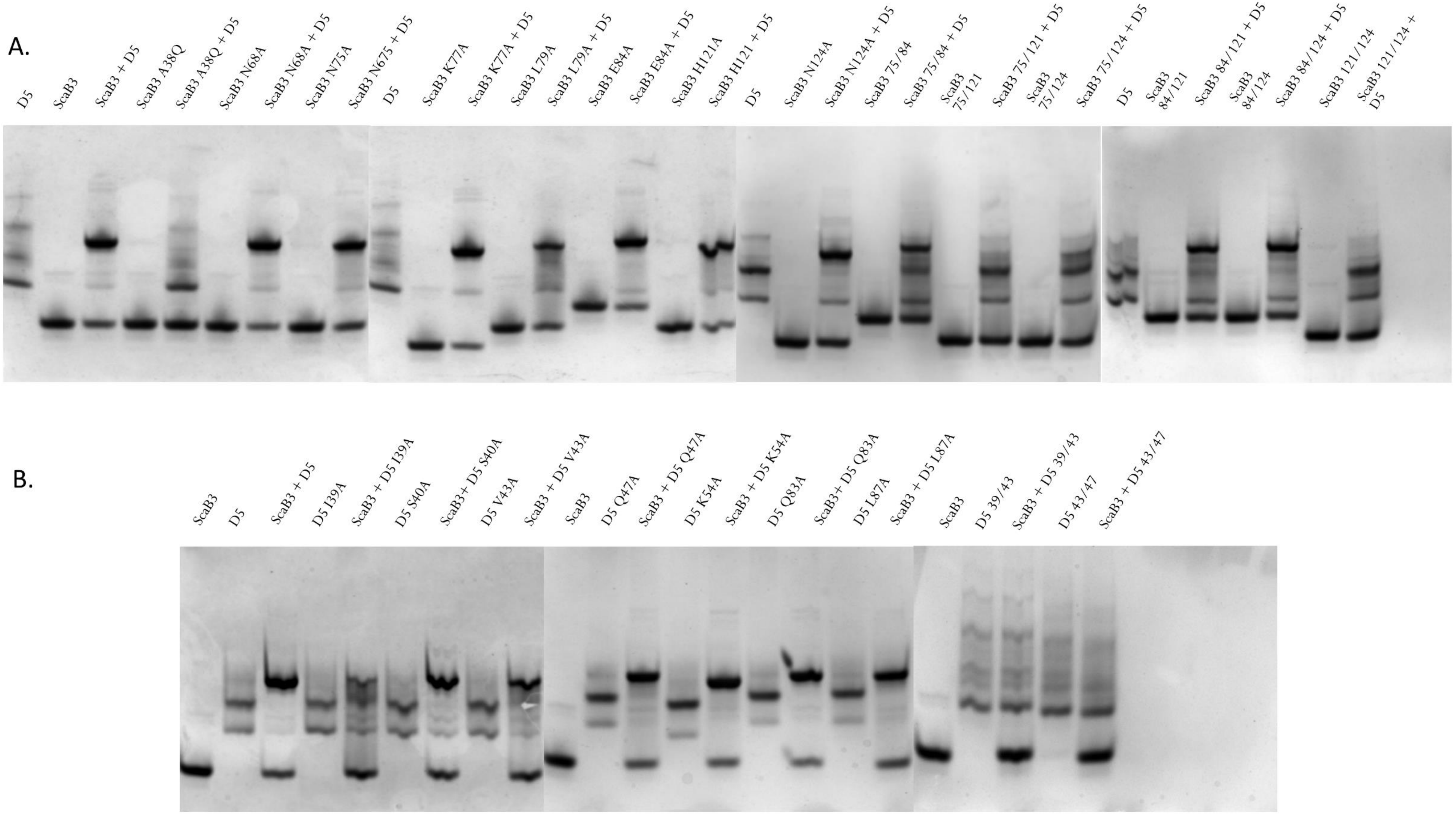
A.
D5
ScaB3
ScaB3 + D5
ScaB3 A38Q
ScaB3 A38Q + D5
ScaB3 N68A
ScaB3 N68A + D5
ScaB3 N75A
ScaB3 N675 + D5
D5
ScaB3 K77A
ScaB3 K77A + D5
ScaB3 L79A
ScaB3 L79A + D5
ScaB3 E84A
ScaB3 E84A + D5
ScaB3 H121A
ScaB3 H121 + D5
D5
ScaB3 N124A
ScaB3 N124A + D5
ScaB3 75/84
ScaB3 75/84 + D5
ScaB3 75/121
ScaB3 75/121 + D5
ScaB3 75/124
ScaB3 75/124 + D5
D5
ScaB3 84/121
ScaB3 84/121 + D5
ScaB3 84/124
ScaB3 84/124 + D5
ScaB3 121/124
ScaB3 121/124 + D5
B.
ScaB3
D5
ScaB3 + D5
D5 I39A
ScaB3 + D5 I39A
D5 S40A
ScaB3+ D5 S40A
D5 V43A
ScaB3 + D5 V43A
ScaB3
D5 Q47A
ScaB3 + D5 Q47A
D5 K54A
ScaB3 + D5 K54A
D5 Q83A
ScaB3+ D5 Q83A
D5 L87A
ScaB3 + D5 L87A
ScaB3
D5 39/43
ScaB3 + D5 39/43
D5 43/47
ScaB3 + D5 43/47

FIGURE S7

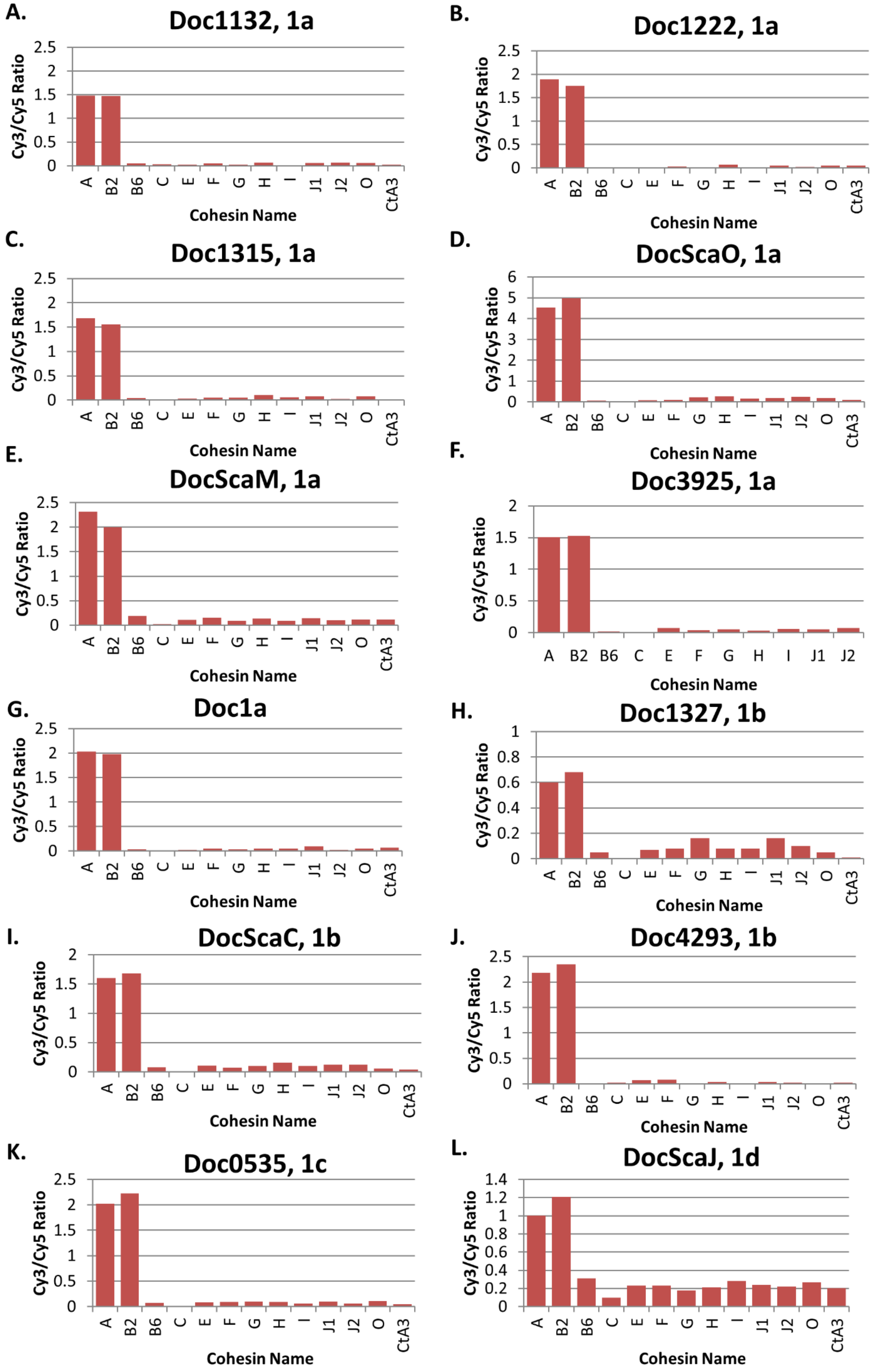
A.
Doc1132, 1a
B.
Doc1222, 1a
C.
Doc1315, 1a
D.
DocScaO, 1a
E.
DocScaM, 1a
F.
Doc3925, 1a
G.
Doc1a
H.
Doc1327, 1b
I.
DocScaC, 1b
J.
Doc4293, 1b
K.
Doc0535, 1c
L.
DocScaJ, 1d
Cy3/Cy5 Ratio
Cohesin Name
A B2 B6 C E F G H I J1 J2 O CtA3

FIGURE S8

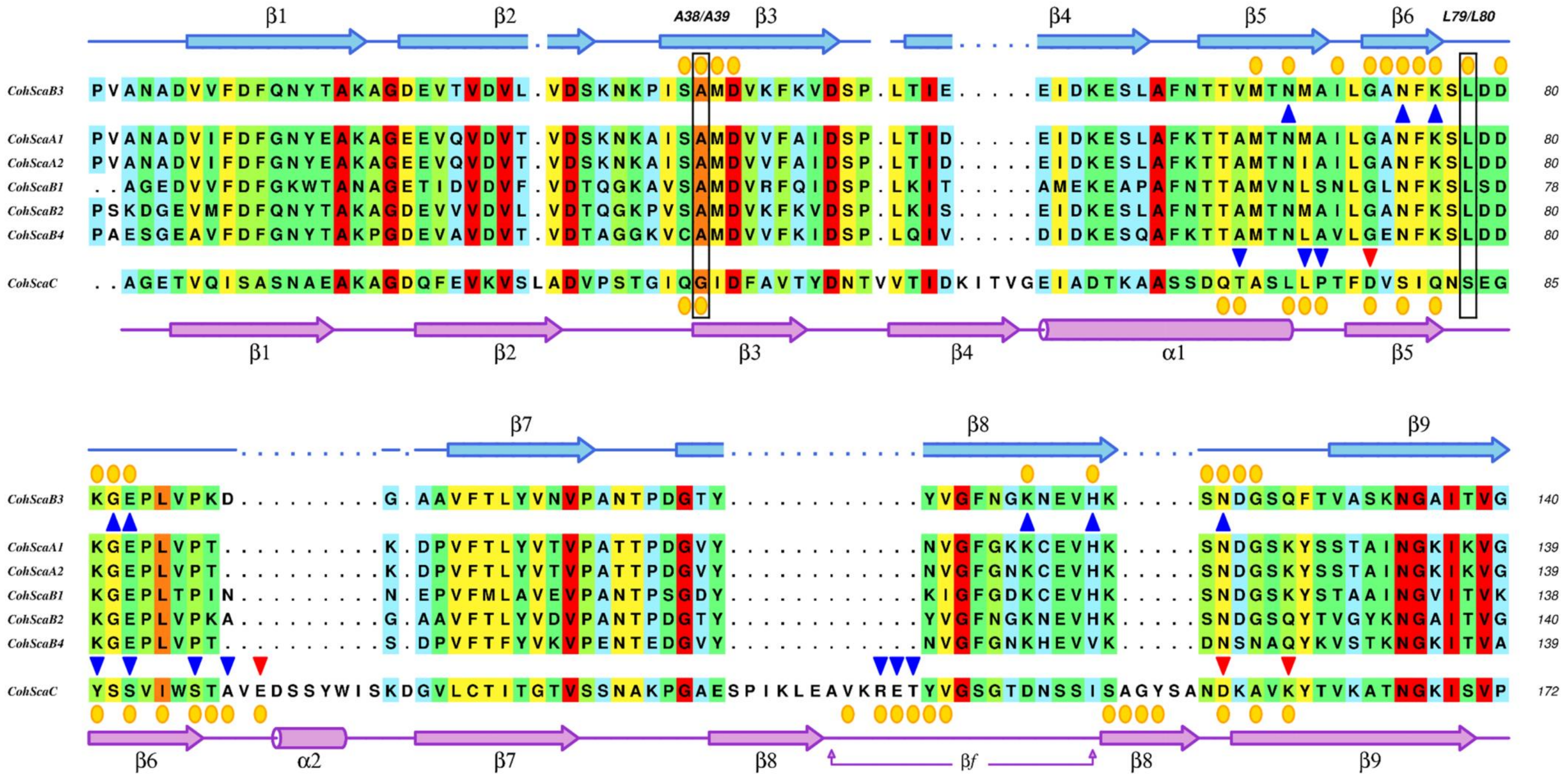

β1 β2 A38/A39 β3 β4 β5 β6 L79/L80
CohScaB3
CohScaA1
CohScaA2
CohScaB1
CohScaB2
CohScaB4
CohScaC
β1 β2 β3 β4 α1 β5
β7 β8 β9
β6 α2 β7 β8 βf β8 β9
